# Integrated metabolomics reveals lipid mediators and food-associated chemicals associated with metabolic syndrome

**DOI:** 10.3389/fnut.2026.1836210

**Published:** 2026-06-22

**Authors:** Chao Shi, Hongjuan Shi, Peifeng Liang, Hong Luan, Ling Ma, Yin Cheng, Lanqiqi Wu

**Affiliations:** 1 People's Hospital of Ningxia Hui Autonomous Region, Ningxia Medical University, Yinchuan, Ningxia Hui Autonomous Region, China; 2 Ningxia Institute of Clinical Medicine, People's Hospital of Ningxia Hui Autonomous Region, Yinchuan, China; 3 School of Public Health, Ningxia Medical University, Yinchuan, Ningxia Hui Autonomous Region, China

**Keywords:** biomarkers, food-associated chemicals, lipid mediators, mediation analysis, metabolic syndrome, metabolomics

## Abstract

**Background:**

Metabolic syndrome (MetS), a cluster of interrelated metabolic abnormalities, poses a major global health burden. However, its underlying molecular pathways and environmental triggers remain incompletely understood. Using an integrated metabolomics approach in a well-characterized Chinese cohort, this study aimed to comprehensively characterize metabolic perturbations associated with MetS and to explore potential mechanistic links with food-related chemical exposures.

**Methods:**

An integrated cross-platform metabolomics strategy was employed in a two-phase study design comprising independent discovery (*n* = 450) and validation (*n* = 450) cohorts. We performed both untargeted and targeted metabolomic profiling on serum samples. In a sub-cohort (*n* = 252), urinary concentrations of eight food-related metals were measured. Analytical workflows encompassed biomarker identification and validation, machine learning-based integration of metabolites to construct diagnostic panels, and quantile g-computation alongside mediation analysis to investigate exposure-metabolite-disease pathways linking food chemicals to MetS.

**Results:**

Eight novel metabolite biomarkers significantly associated with MetS components were identified and validated, including the lipid mediator LPC (20:0) and the food-associated pesticide residue procymidone. Machine learning integration of these biomarkers produced optimized multi-metabolite panels that demonstrated superior diagnostic performance for distinguishing MetS (AUC = 0.791 ± 0.034) and its pre-clinical stages (AUC = 0.864 ± 0.023) from healthy controls. Mediation analysis revealed that LPC (20:0) statistically mediated the observed association between chromium exposure and MetS risk, and procymidone similarly mediated the association between mercury exposure and MetS risk, based on cross-sectional data and this finding requires functional validation.

**Conclusion:**

This study delineates a distinct metabolic signature of MetS. Pending external validation in independent populations, these findings suggest the potential for developing clinically useful diagnostic tools. Furthermore, it provides pioneering evidence that specific metabolites may bridge food-related chemical exposures to systemic metabolic dysregulation, offering an integrated pathophysiological perspective on the role of food in chronic disease.

## Highlights

- This study identifies and validates a panel of eight novel metabolite biomarkers associated with metabolic syndrome (MetS), including the lipid mediator LPC (20:0) and the food-associated pesticide residue procymidone.
- A multi-metabolite panel integrating three biomarkers achieves superior diagnostic performance for MetS (AUC = 0.791 ± 0.034).
- A distinct four-metabolite panel demonstrates efficacy in identifying Pre-MetS individuals (AUC = 0.864 ± 0.023).
- Specific metabolites (LPC (20:0) and Procymidone) are identified as potential mediators linking food-related chemical exposures (chromium/mercury) to MetS risk.
- The findings advance an integrated hypothesis-generating framework connecting environmental exposures to endogenous metabolic dysregulation in MetS, pending validation in independent cohorts.

## 1 Introduction

Metabolic syndrome (MetS) involves a cluster of interconnected metabolic abnormalities, including central obesity, mild dyslipidemia, elevated blood pressure, and impaired glucose tolerance (1). Its clinical significance stems from a strong association with diabetes, stroke, cardiovascular complications and all-cause mortality (2, 3). Globally, MetS prevalence is approximately 24–35% across Europe and Asia, with reported rates of 34.7% in the United States and 33.9% in China (4–7). Despite its high global prevalence, the intricate molecular mechanisms driving MetS progression remain only partially understood, which hampers the development of targeted interception strategies. While its development is known to involve genetic pre-disposition and lifestyle factors (diet, physical inactivity), how these etiological factors translate into molecular dysregulation, particularly through food-related chemical exposures, remains poorly characterized (8–10).

Metabolomics, by providing a functional readout of physiological states through systematic profiling of small molecules, holds unique potential for elucidating MetS mechanisms beyond standard clinical parameters (11–14). Although untargeted, targeted, and lipidomic studies have identified potential MetS biomarkers and metabolic shifts (15–17), they are often limited by modest cohort sizes and methodological shortcomings, such as inadequate control for confounders and lack of blinding, which compromise the generalizability of findings (18). Moreover, existing research has pre-dominantly remained associative, with limited investigation into the etiological factors driving these metabolic changes. Recent meta-analyses have systematically summarized the lipidomic signatures in MetS, confirming the need for well-controlled, two-phase metabolomic investigations to validate candidate biomarkers (19).

Dietary factors play a key role in non-transmissible chronic diseases such as MetS (20). Among these, food lipids, particularly polyunsaturated fatty acids, are fundamental mediators of metabolic alterations (21). Beyond nutrients, humans are exposed to various food-associated chemicals through diet, including pesticide residues and heavy metals that contaminate foodstuffs (22). These food chemical contaminants have been increasingly linked to metabolic dysregulation, yet the underlying metabolic pathways remain largely uncharted (23–25). Elucidating how food-related chemical exposures perturb the human metabolome is a crucial step toward understanding diet-disease relationships.

To bridge this gap, we conducted an integrated study within a well-established cardiovascular screening cohort in Ningxia, China. Our cross-platform metabolomics strategy, combining untargeted and targeted profiling, was designed to map metabolic disturbances in MetS while rigorously controlling for confounders. In a sub-cohort with urinary metal data, we further applied quantile g-computation (qgcomp) and mediation analysis to assess the food chemical mixture's effect on the metabolome and to test specific metabolites as potential mechanistic links between dietary exposures and disease. This comprehensive approach aims to unravel the complex interplay between food-related chemical exposures and metabolic dysregulation in MetS, with the goal of identifying novel biomarkers and intervention pathways.

## 2 Methods

### 2.1 Study design and population

**Figure 1:**
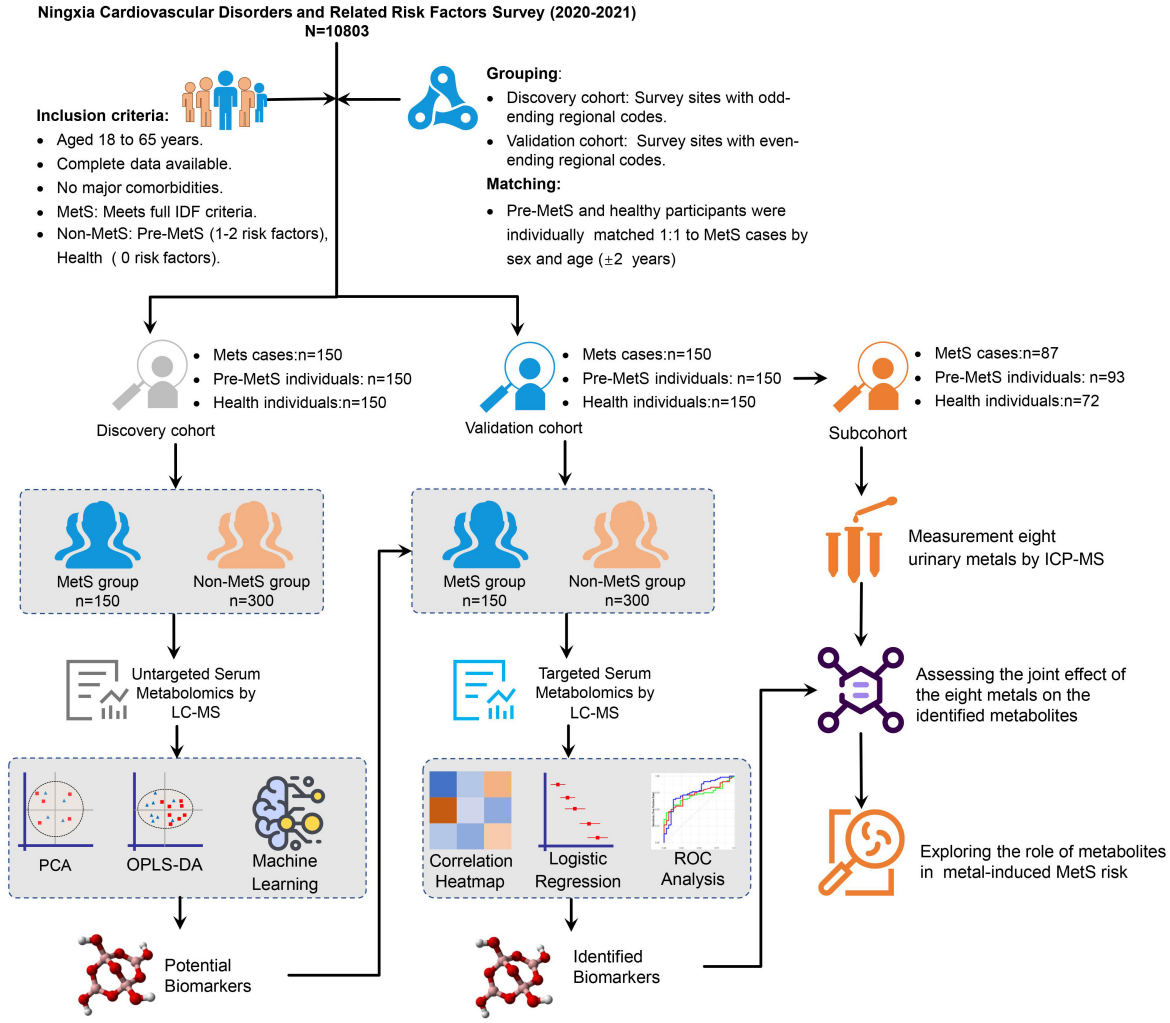
Flowchart of the study design and participant selection.

This analysis used data from the Ningxia Cardiovascular Disorders and Related Risk Factors Survey (NCDS), a population-based study that recruited a regionally representative sample of 10,803 adults between 2020 and 2021 using a four-stage, stratified cluster sampling method (26). From this parent cohort, we randomly selected participants aged 18–65 years who had complete demographic, anthropometric, and laboratory data, and no history of major comorbidities (including cardiovascular disease, cerebrovascular disease, cancer, or end-stage kidney disease). The overall study design and participant flow are depicted in Figure 1.

To establish independent discovery and validation cohorts, participants were partitioned into two groups based on geographic codes (Supplementary Figure S1, Supplementary methods). Based on a power analysis indicating a minimum of 137 subjects per group (Supplementary Figure S2, Supplementary methods), we randomly selected 150 MetS cases from each group. For each case, one healthy control (HC) and one Pre-MetS individual were matched by sex and age (±2 years), yielding a discovery cohort and a validation cohort of 450 participants each (150 per subgroup). From the validation cohort, a pre-defined sub-cohort of 252 participants with previously quantified urinary metal concentrations was used for exposure-metabolism analysis.

### 2.2 Anthropometric and laboratory measurements

All participants were assessed after an overnight fast (≥8 h), including anthropometry and blood pressure measurement. Fasting blood and urine samples were collected. A portion of blood was used for immediate clinical analysis of metabolic parameters [lipids, fasting plasma glucose (FPG), glycated hemoglobin (HbA1c)]. The remaining serum and urine aliquots were stored at −80 °C for subsequent metabolomic and metal analyses. Detailed procedures are in Supplementary methods.

### 2.3 Definition of metabolic status

According to the IDF definition of MetS (27), participants were defined as having MetS if they had abdominal obesity and any two of the following four phenotypes: (1) systolic blood pressure (SBP) ≥ 130 mmHg and/or diastolic blood pressure (DBP ≥ 85 mmHg; (2) triglycerides (TG) ≥ 1.7 mmol/L; (3) FPG ≥ 5.6 mmol/L; or (4) high-density lipoprotein cholesterol (HDL-C) < 1.03 mmol/L in men or < 1.29 mmol/L in women. Abdominal obesity was defined as waist circumference ≥ 90 cm in men and waist circumference ≥ 80 cm in women.

Pre-MetS was defined as the presence of one or two MetS risk factors without meeting the full diagnostic criteria for MetS (28, 29). HC was defined as having no risk factors. For analytical purposes, both pre-MetS and HC groups were combined into a non-MetS group.

### 2.4 Metabolomics profiling

#### 2.4.1 Untargeted metabolomics

Untargeted metabolomic profiling was conducted using ultra-performance liquid chromatography-tandem mass spectrometry (UPLC-MS/MS) with separate chromatographic methods for polar and non-polar metabolites (Supplementary Figure S3, Supplementary methods). Raw data were preprocessed as follows: features with >50% missing values in any group were removed; remaining missing values were imputed with half of the minimum positive value of the corresponding feature. Peak areas were normalized using deuterated isotope-labeled internal standards spiked prior to extraction. Pooled QC samples were injected every 10–12 samples throughout the run to monitor signal drift; features with QC-RSD >30% were excluded. No batch correction was needed because all samples were processed in a single continuous batch. Detailed protocols for sample pre-paration and mass spectrometry are provided in the Supplementary methods.

#### 2.4.2 Targeted metabolomics

Targeted metabolite analysis was performed using a TSQ Access MAX triple quadrupole liquid chromatography-tandem mass spectrometry (HPLC-MS/MS) system. Serum samples were deproteinized with acetonitrile prior to analysis. Complete chromatographic and mass spectrometric parameters are documented in the Supplementary methods (Supplementary Tables S1–3, Supplementary methods).

Detailed cross-validation procedures between untargeted and targeted metabolomics are provided in the Supplementary methods.

### 2.5 Measurement and adjustment for urinary metals

Urinary concentrations of eight metals [As, Cd, chromium (Cr), copper (Cu), mercury (Hg), manganese (Mn), Pb, and zinc (Zn)] were measured by inductively coupled plasma mass spectrometry (ICP-MS). These metals were considered as potential food-related exposures, as diet is a major source for the general population. To account for urine dilution, raw concentrations were standardized using a predicted creatinine value derived from a regression model incorporating age, sex, and anthropometrics. Complete methodological details, including ICP-MS operational parameters, detection limits (Supplementary Table S4) and the standardization formula, are available in the Supplementary methods.

### 2.6 Blinding

To minimize potential bias, a blinding protocol was implemented. Laboratory analyses were performed at two independent sites: untargeted metabolomics and urinary metal measurements were conducted at Shanghai Biotree Biotech, while targeted metabolomic quantification was performed at Ningxia Medical University. Technicians at both sites processed de-identified samples and were blinded to all participant clinical information. Furthermore, during the statistical analysis for biomarker discovery and model building, the analyst was blinded to sample group allocation.

### 2.7 Statistical analysis

Statistical analyses were performed using R software (version 4.4.5). Continuous variables are presented as mean ± standard deviation or median (interquartile range) based on their distribution; categorical variables are presented as percentages. Group comparisons (e.g., HC vs. Pre-MetS vs. MetS) for continuous variables employed one-way ANOVA or Kruskal-Wallis tests, as appropriate, and chi-square tests for categorical variables.

For metabolomics data, we applied a multi-phase analytical strategy. In the discovery phase, orthogonal projections to latent structures-discriminant analysis (OPLS-DA) was performed on untargeted data to identify metabolites most relevant to group separation. Model robustness was assessed by 200-fold permutation testing and CV-ANOVA. Metabolites with a variable importance in projection (VIP) score >2.0 and a univariate *P*-value < 0.05 (Student's *t*-test) were considered candidate dysregulated metabolites. Further refinement was achieved through machine learning-based feature selection using Random Forest (RF) and Support Vector Machine (SVM) algorithms. In the validation and diagnostic phase, the levels of candidate metabolites from targeted quantification were compared across groups using Kruskal–Wallis tests with Bonferroni correction. Spearman's rank correlation was employed to assess associations both among metabolites themselves and between metabolites and MetS (including its individual components). To evaluate the risk contribution of individual metabolites, logistic regression was performed for MetS and each of its components. Finally, we constructed and evaluated diagnostic classifiers using binary logistic regression, RF, and SVM on these validated metabolites, comparing their performance via receiver operating characteristic (ROC) analysis. The optimal model was selected based on the area under the curve (AUC).

To ensure unbiased performance estimation, all diagnostic classifiers were evaluated using a nested 5-fold cross-validation (CV) framework. The entire nested CV procedure was repeated 10 times with different random splits to obtain stable performance estimates. In the outer loop (*k* = 5), the validation cohort was partitioned into five equal folds, in each iteration, four folds served as the training set and one fold as the held-out test set. Hyperparameter optimization was performed exclusively within the training set using an inner 5-fold CV loop. The hyperparameter search grids were as follows: for Random Forest (RF), ntree = {500, 1,000} and mtry = {1, 2, 3, 4}; for Support Vector Machine (SVM) with radial basis function kernel, cost (*C*) = {0.1, 1, 10, 100} and gamma = {0.01, 0.1, 1, 10}; logistic regression used default L2 regularization (*C* = 1) without tuning. Inverse-frequency class weights were applied to address the 1:2 class imbalance present in both groups.

To objectively determine the optimal metabolite panels for the two classification tasks (MetS vs. HC+Pre-MetS and HC vs. Pre-MetS+MetS), we performed a two-stage feature selection procedure within the training folds of the nested cross-validation (i.e., inside the outer training set, before hyperparameter tuning). Specifically, in each inner training fold: (1) RF variable importance (Mean Decrease Gini) was computed using 10 independent model repetitions (ntree = 500 per repetition) on the training data only, and metabolites were ranked by their mean importance score; (2) recursive feature elimination (RFE) based on the Kappa metric was then applied to determine the optimal feature subset size; (3) the one-standard-error rule was used to select the smallest feature set within one standard error of the maximum Kappa value. The feature panels were then locked and used to train the diagnostic classifiers in the inner training folds, with performance evaluated on the corresponding outer test fold. The entire feature selection procedure was repeated within each outer training fold and never accessed the outer test fold, ensuring complete separation of feature selection from performance evaluation.

Performance metrics (AUC, sensitivity, specificity, Brier score) are reported as mean ± standard deviation across all 50 test folds (10 repetitions × 5 outer folds). Calibration curves were additionally employed to assess the agreement between the predicted probabilities and the observed outcomes, thereby evaluating the reliability of the diagnostic models.

To investigate the role of environmental exposures, we first assessed the joint effect of the eight heavy metals on the validated metabolites using qgcomp. This model estimates the overall mixture effect and the weight (direction and magnitude) of each metal's contribution. Subsequently, to explore potential mechanistic pathways, we conducted causal mediation analysis on specific metal-metabolite pairs. Based on the qgcomp results, for each metabolite, the top two contributing metals (by absolute weight) were selected for mediation testing. This analysis quantified the extent to which the association between a given metal exposure and MetS risk was mediated by its associated metabolite, reporting the average causal mediation effect (ACME), average direct effect (ADE), and total effect.

A two-sided *P*-value < 0.05 was considered statistically significant, unless otherwise specified for multiple testing corrections.

## 3 Results

### 3.1 Characteristics of the discovery and validation cohorts

Baseline characteristics of the discovery and validation cohorts are summarized in Table 1. As expected, both cohorts showed a clear graded increase in cardiometabolic risk profiles from the HC to Pre-MetS to MetS groups, with significant stepwise elevations in measures of glycemia (HbA1c, FPG) and dyslipidemia (TG, TC, LDL-C; all between-group comparisons *P* < 0.05). Sex distribution was balanced across groups within each cohort. The two cohorts were largely comparable in key MetS-related characteristics (Supplementary Table S5, Supplementary results). However, they differed significantly in age and smoking history (both *P* < 0.05).

**Table 1:** Baseline characteristics of study participants stratified by metabolic syndrome status in the discovery and validation cohorts

Variables	Total(*N* = 900)	Discovery cohort			*P-*value	Validation cohort			*P*-value
		MetS (*n* = 150)	Pre-MetS (*n* = 150)	HC (*n* = 150)		MetS (*n* = 150)	Pre-MetS (*n* = 150)	HC (*n* = 150)	
Gender, *n* (%)									
Male	420 (46.47)	73 (48.67)	73 (48.67)	73 (48.67)	1.000^1^	67 (44.67)	67 (44.67)	67 (44.67)	1.000^1^
Female	480 (53.33)	77 (51.33)	77 (51.33)	77 (51.33)		83 (55.33)	83 (55.33)	83 (55.33)	
Age, median (quartile)	39 (32; 48)	37 (32, 45)	38 (33, 47)	38 (31, 45)	0.024^2^	40 (33, 51)	39 (32, 51)	40 (32, 51)	0.650^2^
Smoking, *n* (%)	243 (27.00)	50 (33.33)	36 (24.00)	49 (32.67)	0.144^1^	39 (26.00)	34 (22.67)	35 (23.33)	0.774^1^
Alcohol consumption, *n* (%)	227 (25.22)	43 (28.67)	36 (24.00)	46 (30.67)	0.417^1^	39 (26.00)	27 (18.00)	36 (24.00)	0.227^1^
Waist Circumference, mean ± SD	82.65 ± 11.08	93.23 ± 8.74	81.24 ± 9.38	74.29 ± 6.48	<0.001 ^3^	92.60 ± 7.69	80.11 ± 8.08	74.44 ± 7.11	<0.001^3^
BMI, *n* (%)									
<24.0	396 (44.00)	10 (5.03)	66 (44.00)	123 (82.00)	<0.001^1^	6 (4.00)	75 (50.00)	116 (77.33)	<0.001^1^
24.0–27.9	298 (33.11)	58 (38.67)	64 (42.67)	26 (17.33)		59 (39.33)	57 (38.00)	33 (22.00)	
≥28.0	206 (22.89)	82 (54.67)	20 (13.33)	1 (0.67)		85 (56.67)	18 (12.00)	1 (0.67)	
Hypertension, *n* (%)	345 (38.33)	107 (71.33)	56 (37.33)	0 (0.00)	<0.001^1^	111 (74.00)	71 (47.33)	0 (0.00)	<0.001^1^
Hyperglycemia, *n* (%)	292 (32.44)	93 (62.00)	58(38.67)	0 (0.00)	<0.001^1^	88 (58.67)	53 (35.33)	0 (0.00)	<0.001^1^
HbA1c (%), mean ± SD	5.58 ± 0.03	5.88 ± 0.08	5.47 ± 0.04	5.38 ± 0.03	<0.001^3^	5.84 ± 1.09	5.48 ± 0.72	5.32 ± 0.32	<0.001^3^
FPG (mmol/l), mean ± SD	5.58 ± 0.07	6.23 ± 0.17	5.52 ± 0.07	5.00 ± 0.04	<0.001^3^	6.20 ± 2.00	5.58 ± 1.58	5.03 ± 0.36	<0.001^3^
TG (mmol/l), mean ± SD	1.60 ± 0.80	2.41 ± 0.15	1.54 ± 0.17	0.86 ± 0.03	<0.001^3^	2.19 ± 1.64	1.32 ± 0.97	0.88 ± 0.28	<0.001^3^
TC (mmol/l), mean ± SD	4.21 ± 0.04	4.46 ± 0.08	4.11 ± 0.07	4.06 ± 0.06	0.026^3^	4.33 ± 0.96	4.11 ± 0.85	4.14 ± 0.81	0.727^3^
HDL-C (mmol/l), mean ± SD	1.29 ± 0.01	1.15 ± 0.02	1.26 ± 0.02	1.47 ± 0.02	<0.001^3^	1.10 ± 0.24	1.23 ± 0.26	1.45 ± 0.23	<0.001^3^
LDL-C (mmol/l), mean ± SD	2.51 ± 0.03	2.72 ± 0.07	2.49 ± 0.06	2.34 ± 0.05	0.342^3^	2.69 ± 0.76	2.49 ± 0.66	2.40 ± 0.82	0.309^3^

MetS: metabolic syndrome; Pre-MetS: Pre-metabolic syndrome; HC: health control; BMI, body mass index; HbA1c, glycated hemoglobin; FPG, fasting plasma glucose; TG, triglycerides; TC, total cholesterol; HDL-C, high-density lipoprotein cholesterol; LDL-C, low-density lipoprotein cholesterol.

1 P-values were obtained from chi-square test.

2 P-value was obtained from Kruskal–Wallis rank-sum test.

3 P-values were obtained from Oneway ANOVA.

### 3.2 Discovery of differential metabolites via untargeted profiling

Untargeted metabolomic profiling of the discovery cohort serum samples was performed following rigorous quality control, as evidenced by principal component analysis (PCA) showing most samples within the 95% confidence interval (Supplementary Figure S4, Supplementary results). OPLS-DA analysis revealed clear metabolic separation between the MetS and non-MetS Supplementary groups (Supplementary Figures S5A, B, Supplementary results). The model was robust, as confirmed by permutation testing (200 permutations, Supplementary Figure S5C) and CV-ANOVA (*P* < 0.0001, Supplementary Table S6), with no evidence of overfitting. Using criteria of VIP > 2.0 and *P* < 0.05, we identified 208 differentially abundant metabolites (Supplementary Table S7, Supplementary results). Subsequent machine learning-based feature selection using RF and SVM refined this list to 29 high-confidence candidate biomarkers for further validation (Table 2). Their dysregulation patterns (17 upregulated, 12 downregulated) are shown in the volcano plot (Supplementary Figure S6, Supplementary results). Detailed identification parameters are given in Supplementary Table S8 in Supplementary results. KEGG pathway enrichment analysis using MetaboAnalyst 6.0 on the 208 differential metabolites revealed significant enrichment (*P* < 0.0001) in multiple pathways, including “Valine, leucine and isoleucine biosynthesis”, “Glycine, serine and threonine metabolism”, “Alanine, aspartate and glutamate metabolism”, “ABC transporters”, and “Central carbon metabolism in cancer”. The results are presented as a bubble chart and a classification bar chart (Supplementary Figures S7, S8, Supplementary results).

**Table 2:** List of differential metabolites identified in the untargeted metabolomic analyses.

Metabolite	RT (min)	m/z	FC	VIP	*P*-value
Benzoyleneurea	252.5	161.0363	0.954	2.164	2.28E-08
1-Methylguanosine	204.8	298.1131	1.493	2.469	1.08E-07
Nonaethylene glycol	68.5	413.2395	1.250	2.050	5.80E-09
2,4-Dihydroxybutanoic acid	305.3	101.0244	1.273	2.397	1.95E-11
Indole-3-pyruvic acid	57.5	202.051	1.253	1.146	<0.001
S-Methylmethionine	74.2	147.0758	1.088	1.423	<0.001
(R)-3-Amino-3-(3-chlorophenyl) propionic acid	256	198.033	1.101	2.230	3.58E-09
N-Methylvaline	66.5	132.1011	1.190	2.219	1.63E-13
1,2,2,6,6-Pentamethyl-4-piperidinol	431.5	172.1684	0.901	1.450	5.27E-05
4-(Chloromethyl)-7-hydroxy-8-methyl-2H-chromen-2-one	40.8	225.0331	1.138	1.803	3.51E-10
2-Imino-1-imidazolidineacetic acid	280.5	142.0611	1.051	1.839	5.83E-06
2-Hydroxydesmethylimipramine	29.8	283.1825	1.116	1.046	7.27E-08
Oxacillin	209.3	420.0596	1.120	1.307	5.48E-08
4,5-Dihydro-2-methylthiazole	205.6	102.0335	0.887	1.424	9.03E-05
SM (d18:1/17:0)	201.6	717.5891	0.438	2.174	2.57E-24
1,3,5-Trimethyl-2,4,6-tris (3,5-di-tert-butyl-4-hydroxybenzyl) benzene	201.1	773.5812	0.410	2.777	2.04E-21
3,7,8-Trihydroxy-3-methyl-10-oxo-1,4-dihydropyrano[4,3-b]chromene-9-carboxylic acid	196.6	307.0517	0.712	2.283	2.61E-11
SM (d18:1/20:0)	199.4	759.6341	0.368	3.051	9.50E-28
Biliverdin	426	581.2435	0.736	1.166	1.09E-07
2,6-Di-tert-butylphenol	495.5	205.1605	0.933	2.861	9.29E-17
Methyl 2-(4-chlorophenyl) acetate	43.4	185.0407	1.914	2.535	3.52E-09
Pyridine	54.6	80.0490	0.914	2.483	8.59E-17
Phytanic acid	34.1	311.2957	0.589	2.278	7.72E-18
LPC (20:0)	210.1	552.4005	0.641	2.367	1.06E-23
Procymidone	129.4	282.0093	1.267	2.024	4.52E-16
3,4-Thiophenedicarbonitrile	46.5	135.0022	1.847	1.259	2.05E-15
Tafluprost	360.8	453.2413	2.681	2.165	4.73E-14
7,12-Dioxolithocholic acid	406.5	387.251	1.737	1.720	7.83E-14
N-Methyl-D-aspartic acid	301.7	146.0459	1.630	1.220	5.02E-10

RT, retention time; m/z, mass-to-charge ratio; FC, fold change (HC/MetS); VIP, variable importance in projection.

### 3.3 Validation of differential metabolites by targeted metabolite analysis

**Figure 2:**
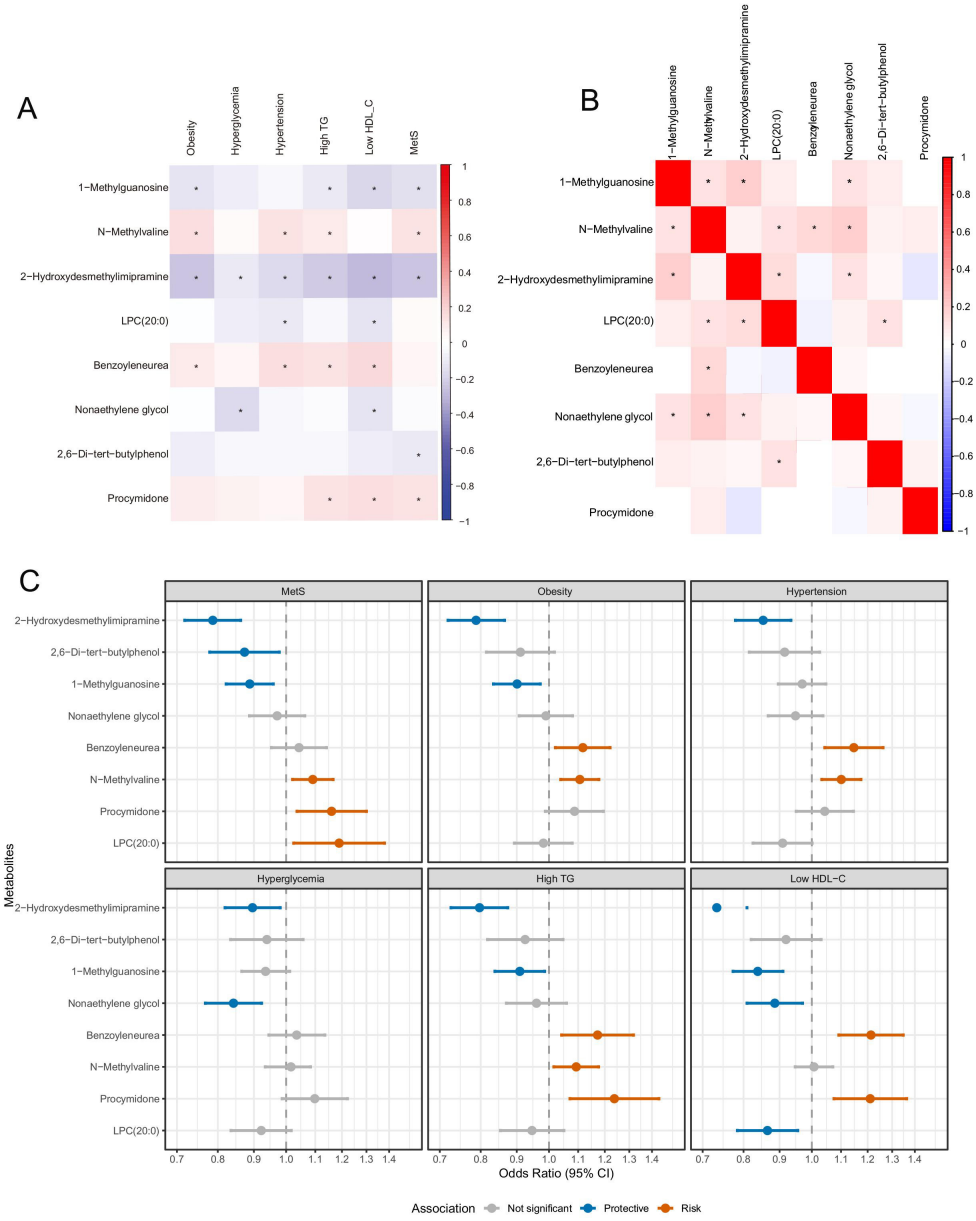
Associations and Risk of Candidate Metabolites with Metabolic Syndrome and Its Components. **(A)** Correlations of the eight candidate metabolites with both MetS and its individual components. **(B)** Correlation heatmap among the eight candidate metabolites. **(C)** Odds ratios for the associations of the eight metabolites with the risk of MetS and its individual components after adjustment for age and sex. MetS, metabolic syndrome.

To validate the 29 candidate biomarkers, we developed a targeted HPLC-MS/MS method. Of the 13 metabolites with available standards, eight were reliably quantified and thus validated (Table 3; method validation data in Supplementary Tables S9, 10 and Supplementary Figure S9, Supplementary results), including the lipid mediator LPC (20:0) and the food-associated pesticide residue procymidone. The TIC chromatograms of the targeted runs under two elution conditions are shown in Supplementary Figure S10 (Supplementary results). Each metabolite correlated with at least one clinical component (Figure 2A). Figure 2B presents a correlation heatmap among the metabolites, revealing distinct clusters of positive and negative interrelationships. After adjusting for age and sex, six metabolites showed significant associations with overall MetS risk (Figure 2C): 2-Hydroxydesmethylimipramine, 2,6-Di-tert-butylphenol, and 1-Methylguanosine were positively associated with risk, while N-Methylvaline, procymidone, and LPC (20:0) were inversely associated. Furthermore, dose–response analysis demonstrated that, with the exception of Procymidone, the remaining seven metabolites displayed significant dose–response relationships within the MetS group (Supplementary Figure S11, Supplementary results).

**Table 3:** Regression equation, correlation coefficient, linear range and limit of quantification for eight Metabolites in the targeted metabolomic analyses.

Metabolite	Regression equation	*R* ^2^	Linear range (ng/mL)	Limit of quantitation (pg)
N-Methylvaline	*y* = 0.2329x + 349.0636	0.9995	4.92–1,180.02	245.84
LPC (20:0)	*y* = 0.0001x + 0.0446	0.9962	62.41–1,248.20	3,120.50
1-Methylguanosine	*y* = 0.0001x + 0.0617	0.9964	18.45–1,476.24	922.65
2-Hydroxydesmethylimipramine	*y* = 0.00176x + 0.9077	0.9967	5.00–1,200.19	250.04
2,6-Di-tert-butylphenol	*y* = 0.0008x + 0.9296	0.9950	6.64–1,592.65	331.80
Nonaethylene glycol	*y* = 0.0006x + 0.5184	0.9992	16.31–652.30	815.38
Benzoyleneurea	*y* = 0.00009x + 0.0868	0.9970	5.28–1,266.20	263.79
Procymidone	*y* = 0.0007x + 2.1543	0.9973	61.63–1,232.60	3,081.49

### 3.4 Evaluation of the diagnostic and predictive performance of the biomarkers

**Figure 3:**
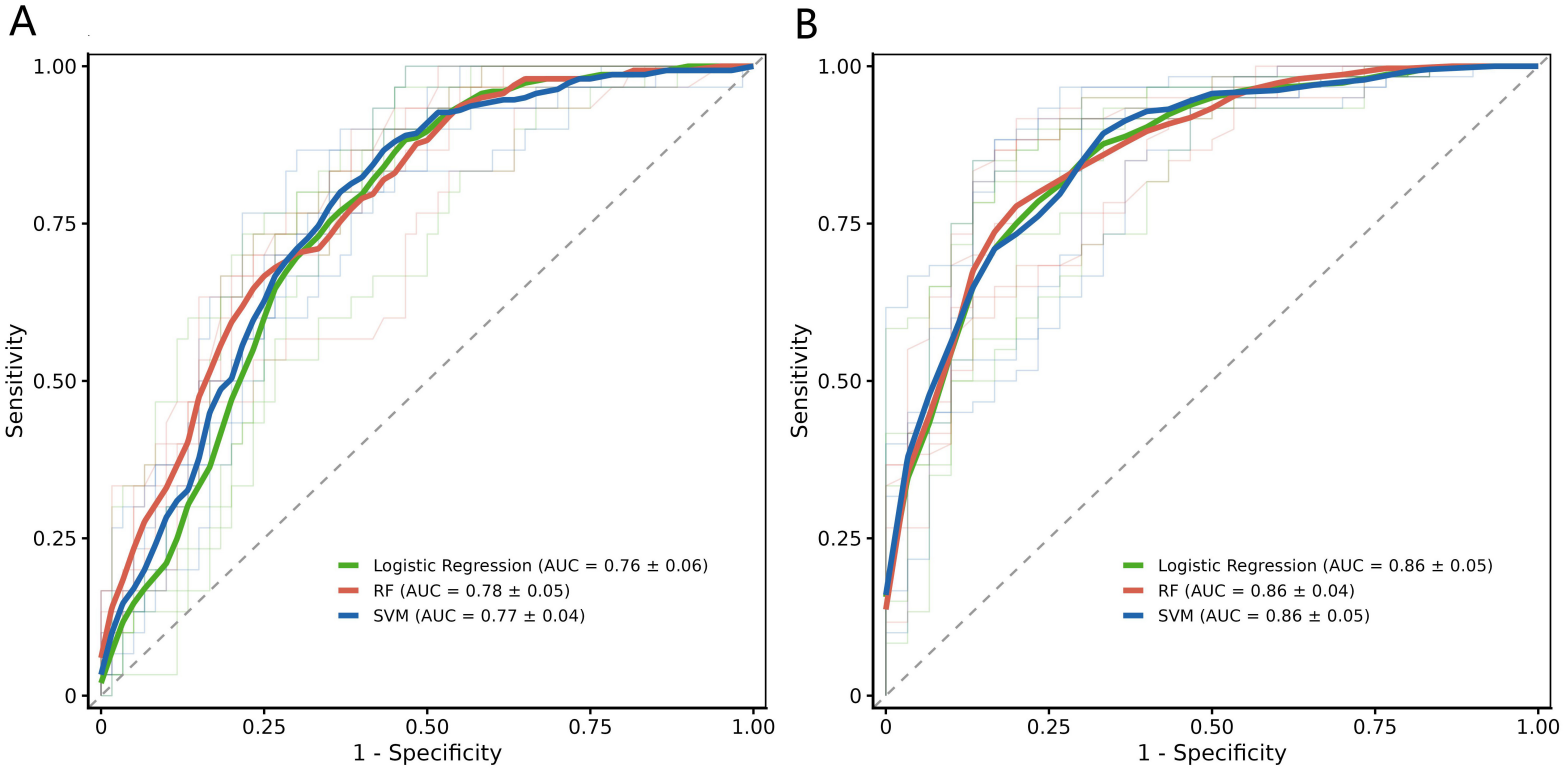
The diagnostic performance of the metabolite classifier in the validation cohort. **(A)** The ROC curve of the metabolite classifier including 2-Hydroxydesmethylimipramine, N-Methylvaline, and LPC (20:0) for diagnosing MetS. **(B)** The ROC curve of the metabolite classifier including LPC (20:0), 1-Methylguanosine, N-Methylvaline, 2-Hydroxydesmethylimipramine for predicting Pre-MetS. MetS, metabolic syndrome; Pre-MetS: pre-metabolic syndrome; ROC, receiver operating characteristic.

ROC analysis revealed limited diagnostic efficacy of individual metabolites for MetS (Supplementary Figure S12, Supplementary results), prompting the construction of diagnostic multi-metabolite panels for MetS and its progression. We first performed a two-stage feature selection included RF variable importance followed by recursive feature elimination, (Supplementary Figure S13, Supplementary results). Three metabolites [2-Hydroxydesmethylimipramine, N-Methylvaline, LPC (20:0)] were selected for distinguishing MetS from HC and Pre-MetS group, and four metabolites (LPC (20:0), 1-Methylguanosine, N-Methylvaline, 2-Hydroxydesmethylimipramine) for discriminating HC from the Pre-MetS and MetS group. ROC curves for the best-performing models are shown in Figures 3A, B. For the MetS vs. HC+pre–MetS group, RF achieved the highest AUC (0.791 ± 0.034), followed by SVM (0.786 ± 0.019) and logistic regression (0.757 ± 0.033), for the HC vs. Pre-MetS+MetS group, both RF (AUC = 0.864 ± 0.023) and SVM (AUC = 0.860 ± 0.032) performed excellently, with logistic regression showing comparable discrimination (AUC = 0.859 ± 0.030; Supplementary Tables S11, Supplementary results). The feature panels were selected using only the training folds of the nested cross-validation; the test folds were held out until final evaluation, thus preventing information leakage. Calibration curves were additionally employed to assess the agreement between predicted probabilities and observed outcomes, which indicated good concordance across all three classifiers (Supplementary Figure S14, Supplementary results). All classifiers were trained using a nested cross-validation framework that performed hyperparameter optimization within the inner loop, and the result showed stable discriminative performance across outer test folds, with fold-wise AUC values showing low variability (Supplementary Table S12, Supplementary results).

### 3.5 Mediation analysis: Exploring the role of metabolites in metal-induced MetS risk

**Figure 4:**
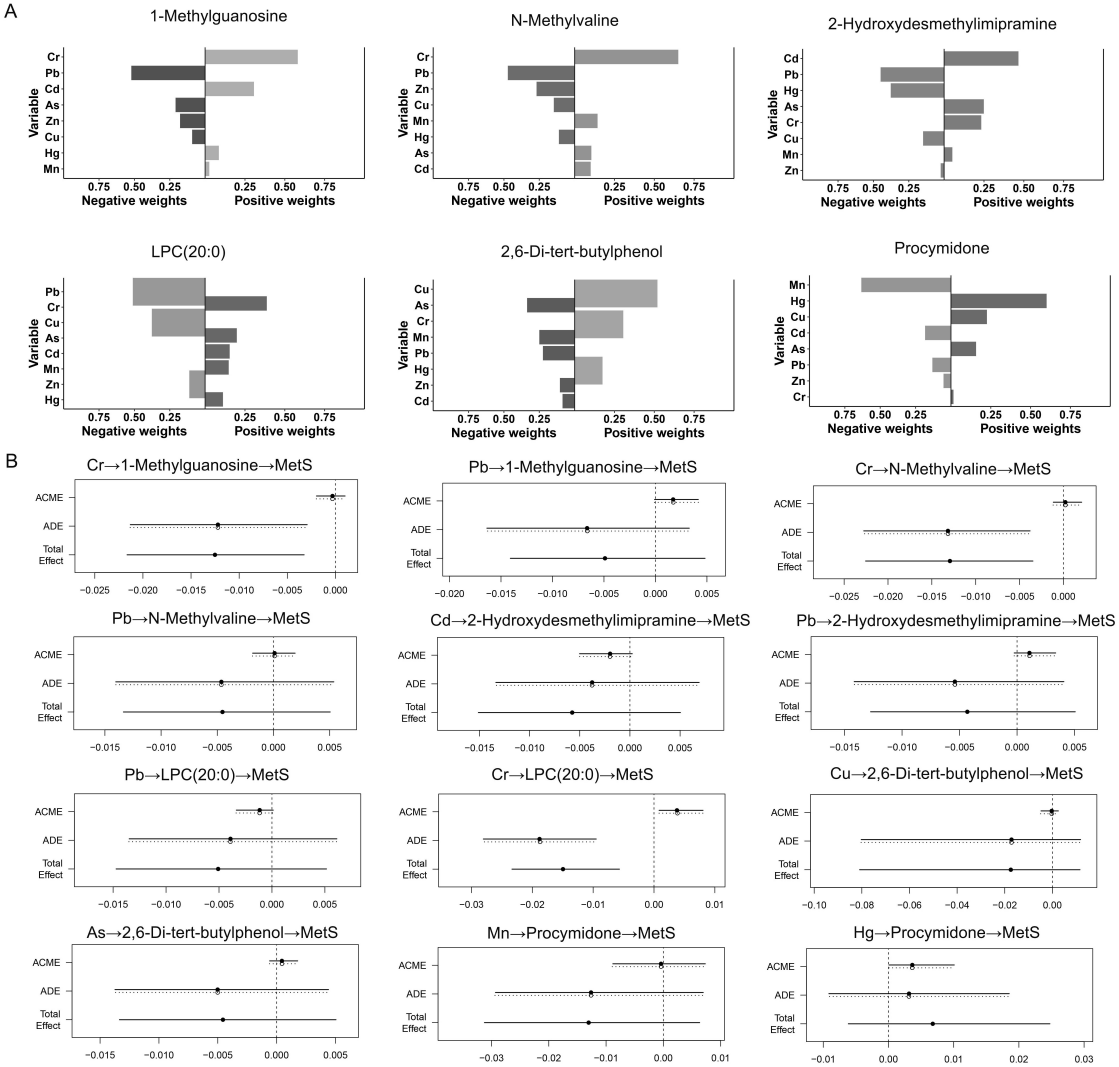
Joint and mediated effects of urinary metals on MetS-associated metabolites. **(A)** Assessment of the overall joint effect of the metal mixture on six MetS-related metabolites using quantile g-computation. **(B)** Metal-specific weights from quantile g-computation models and the subsequent identification of significant mediation pathways linking specific metals to MetS via metabolites.

Qgcomp analysis was applied to the validation sub-cohort (*n* = 252) to assess the joint effect of eight urinary metals on the six MetS-associated metabolites (Figure 4A; sub-cohort characteristics in Supplementary Table S13, Supplementary results). The overall mixture effect was not statistically significant. However, examination of metal-specific weights (Figure 4B) allowed identification of individual metals with the strongest marginal contributions for further mediation analysis. This mediation analysis identified two significant pathways: LPC (20:0) mediated the association between chromium exposure and MetS risk, and procymidone mediated the association between mercury exposure and MetS risk. Complete mediation results for all tested pathways, including point estimates, 95% confidence intervals, and proportion mediated, are provided in Supplementary Table S14 in Supplementary results. These associations should be interpreted as hypothesis-generating and do not imply causation or confirmed biological activity.

## 4 Discussion

This integrated metabolomics study advanced the understanding of MetS on three interconnected fronts: by identifying a panel of novel and paradoxical metabolite biomarkers, establishing the superior diagnostic value of multi metabolite panels derived via machine learning, and pioneering initial evidence for specific metabolites mediating the link between food-related chemical exposures and MetS risk. These findings collectively move beyond associative observations to offer a more integrated pathophysiological view of MetS, in which endogenous metabolic dysregulation interfaces with modifiable dietary factors.

Our metabolomic profiling identified a set of novel biomarkers that functionally stratified MetS risk. These molecules extend beyond traditional risk indicators and included species with protective, risk enhancing, and context dependent associations. For instance, N-methylvaline exhibited a strong risk association particularly with obesity and hypertension, while 2-hydroxydesmethylimipramine, a metabolite of imipramine, was linked to protective effects, a finding that contrasted with the known metabolic risks of the parent drug.

The biological implications of these metabolites point to distinct and plausible mechanistic pathways. The association of N-methylvaline, a methylated branch chain amino acid derivative, with obesity and hypertension aligned with pathways involving chronic mTOR activation and endoplasmic reticulum stress, suggesting it may be an active contributor to metabolic deterioration rather than a passive marker (30–32). The importance of branched-chain amino acid metabolism is further supported by our KEGG enrichment analysis, which identified the “Valine, leucine and isoleucine biosynthesis” pathway as the most significantly perturbed, indicating a systemic disruption of amino acid homeostasis in MetS. Conversely, the protective role of 2-hydroxydesmethylimipramine highlighted the critical, yet often overlooked, principle that a drug and its metabolites can have divergent biological impacts, urging metabolite-specific assessment of pharmacological effects (33–35). Most intriguing was the dual role of LPC (20:0), a monoacyl phosphatidylcholine that serves as an essential cell membrane component and plays a significant role in lipoprotein metabolism (36, 37). While positively associated with overall MetS risk, it was inversely linked to low HDL-C in our study. This paradox likely reflects the pleiotropic nature of lipid species. The same molecule may engage in pro-inflammatory pathways while simultaneously supporting reverse cholesterol transport. Thus, LPC (20:0) may serve as an indicator of competing metabolic fluxes.

A key finding of this study is the identification of food-related chemicals within the metabolic signature of MetS. LPC (20:0), a lysophosphatidylcholine derived from dietary phospholipids, reflects the metabolic fate of ingested fatty acids and may serve as a link between dietary lipid intake and MetS pathogenesis. Procymidone, a fungicide widely used in agriculture, represents a class of food-associated chemical contaminants. Its association with MetS risk suggests that pesticide residues, beyond traditional nutrients, may contribute to metabolic dysregulation. These findings highlight the need to consider both food lipids and food chemical contaminants in understanding diet-related chronic diseases.

Notably, our analysis also detected exogenous compounds, such as the fungicide procymidone, within the metabolic signature of MetS. This finding offers preliminary, cross-sectional evidence that environmental chemicals are detectable within the host metabolome and that their levels are associated with metabolic dysregulation; however, whether they directly intersect with or perturb the metabolome requires functional validation. It moved the concept of environmental contribution to MetS beyond indirect association, raising the hypothesis that these compounds or their metabolic byproducts could be linked to the disease-related metabolic network. Causal inference and mechanistic understanding await further experimental studies. Similarly, nonaethylene glycol (a synthetic polyethylene glycol derivative used in manufacturing and pharmaceuticals) was detected among the eight differential metabolites but was not selected into the final SVM panels; its presence likely reflects medication excipients or environmental exposure, not contamination (blank controls negative) (38). Recent meta-analyses have substantiated the link between pesticide residues (including fungicides) and metabolic disorders, as well as between heavy metal exposures and MetS risk (39, 40).

Given the inherent diagnostic limitation of any single metabolite, we employed machine learning to integrate these multifarious signals into a multi-metabolite panel. This panel demonstrated superior performance in distinguishing not only MetS patients but also individuals with Pre-MetS from healthy controls. This validates the necessity of a multi-factor integration strategy to capture the systemic and heterogeneous nature of MetS (41). The success of this model implied that the combined pattern of these metabolites, reflecting inflammation, lipid dysregulation, microbial co-metabolism, and food-related chemical exposure, provided a more robust signature of pathological state than any single pathway. This offered a proof-of-concept for developing metabolomics-based tools for early risk stratification.

A key mechanistic advance of this study was the elucidation of specific pathways linking food-related exposures to metabolic dysregulation. While the overall mixture effect of heavy metals on the metabolome was non-significant, a result potentially due to antagonistic interactions between pro-oxidant metals like Cd and antioxidant elements like Zn, targeted mediation analysis revealed discrete toxicological pathways. Specifically, we found that Cr exposure might contribute to MetS risk partly through elevating LPC (20:0). Given the dual role of LPC (20:0), this suggested Cr may disrupt lipid homeostasis in a manner that simultaneously increased general risk while paradoxically influencing HDL-C metabolism. More directly, Hg appeared to exert risk through association with procymidone, implying that this heavy metal might influence metabolic health by modulating the persistence, metabolism, or biological activity of pervasive agricultural chemicals. These findings translated broad dietary associations into testable mechanistic hypotheses, proposing that food-associated chemicals act not only through generic oxidative stress but also by hijacking specific endogenous and xenobiotic metabolic circuits.

The strengths of this study included its focus on an understudied population, its coverage of participants across the full MetS progression spectrum, and a rigorous two-phase design encompassing independent discovery and validation conducted on distinct analytical platforms. A key methodological advantage was provided by employing separate biological matrices: serum for metabolomic profiling and urine for food-related metal assessment. This approach minimized the potential for technical confounding.

However, several limitations had to be acknowledged. The cross-sectional design precludes causal inference; mediation effects are hypothesis-generating statistical associations requiring longitudinal validation. Exogenous compound detection (e.g., procymidone) does not imply biological activity; our mediation findings require functional validation before mechanistic interpretation. Second, the analysis was limited to metabolomic data, and future multi-omics studies would be needed to elucidate underlying mechanisms. Third, the small urinary metal sub-cohort (*n* = 252) limited statistical power. Given the small effect sizes (ACME/ADE: −0.03 to 0.03), these mediation results are exploratory and warrant caution. Fourth, food chemical exposure data were available only for a subset, limiting generalizability. Moreover, no direct dietary assessment (e.g., food frequency questionnaires) was performed, making it difficult to disentangle whether metabolomic changes reflect diet, environmental contamination, or both. Furthermore, urinary metals may originate from occupational, air, water, or soil sources rather than diet alone; therefore, our use of ‘food-associated' is hypothesis-generating and does not imply proven dietary attribution. Several residual confounders (dietary intake, physical activity, socioeconomic status, and current medication use) could not be addressed due to lack of data collection, and their potential influence on metabolite profiles and MetS risk should be considered when interpreting our findings. Finally, both discovery and validation cohorts were drawn from the same Ningxia region, limiting external generalizability. Moreover, the fungicide procymidone depends on local agricultural practices. Therefore, our findings, particularly the metabolite panels and food chemical associations, require validation in other populations.

## 5 Conclusions

This study identifies eight metabolite biomarkers of MetS, including LPC (20:0) and the pesticide procymidone. Biologically, they implicate disrupted amino acid and lipid metabolism. Statistically, a three-metabolite panel (AUC = 0.791 ± 0.034) and a four-metabolite panel (AUC = 0.864 ± 0.023) were validated by nested cross-validation. Clinically, these panels represent candidate blood-based early risk stratification tools that require external validation before they can be considered for clinical application. Furthermore, we generated hypothesis-forming evidence that LPC (20:0) and procymidone may link food-associated chemical exposures to MetS risk in this Ningxia cohort. Future external validation, functional studies, and intervention trials are required to establish causality and generalizability. These findings collectively advance an integrated framework for understanding MetS pathophysiology, highlighting the role of food-related exposures in chronic disease.

## Data Availability

The original contributions presented in the study are included in the article/supplementary material, further inquiries can be directed to the corresponding author.
