# Supplementary material for: Integrated metabolomics reveals lipid mediators and food-associated chemicals associated with metabolic syndrome
Source: Front Nutr. 2026 Jun 22;13:1836210. doi: 10.3389/fnut.2026.1836210 (PMC13333618; doi:10.3389/fnut.2026.1836210)
Supplement: Supplementary file 1 [file Data_Sheet_1.pdf]

Supplementary Methods

Selection process of the participants and sample size calculation

Initially, the nine counties included in the Ningxia Cardiovascular Disorders and Related Risk Factors Survey (NCDS) were divided into two cohorts based on the parity of the final digit in their administrative codes. Counties with odd-ending codes were assigned to the odd-numbered group, while those with even-ending codes were allocated to the even-numbered group (Figure S1).

To ensure sufficient statistical power, the minimum required sample size was determined using PASS software. Key input parameters included: three comparison groups (HC, pre-MetS, and MetS), estimated within-group standard deviations, a significance level of 0.05, and a power of 80%. Based on these criteria, the software calculated that a minimum of 137 participants per group was necessary to detect the anticipated effect sizes under the specified statistical conditions (Figure S2). Considering practical feasibility, 150 MetS cases were targeted for inclusion.

In accordance with the study's predefined eligibility criteria, 150 MetS participants were randomly selected from the odd-numbered group to form the case group for the discovery cohort. For each MetS case, one healthy control (HC) and one pre-MetS control were matched on gender and age (±2 years), resulting in a discovery cohort of 450 individuals (150 per subgroup: MetS, pre-MetS, HC). An identical selection and matching procedure was applied to the even-numbered group to construct the validation cohort (n=450), maintaining consistent case numbers and matching criteria between the two cohorts.

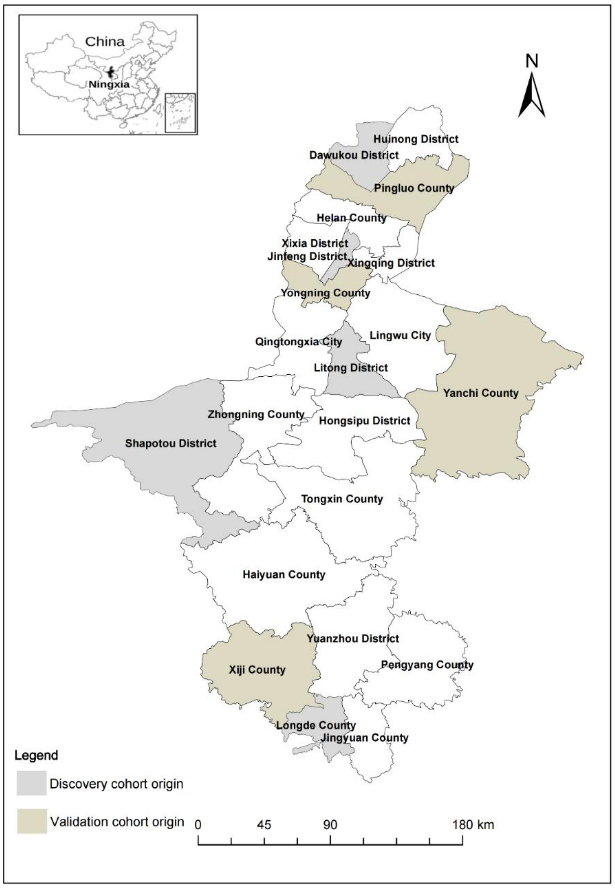

Figure S1 Distribution of study area for cardiovascular disorders and the related risk factors survey in Ningxia Hui Autonomous Region, 2020-2021.

PASS 2023, v23.0.2

One-Way Analysis of Variance Assuming Equal Variances (F-Tests)

Numeric Results

Solve For: Sample Size
Number of Groups: 3

	Sample Size		Means				
Power	Total N	Grp ni	Means μ1	SD σm1	Std Dev σ	Effect Size σm1/σ	Alpha
0.80073	411	137	μ1(1)	82.902	539	0.154	0.05

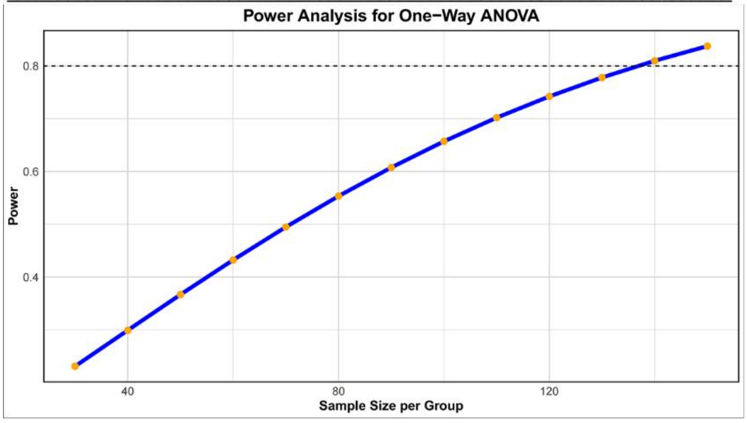

Figure S2 Power analysis. A sample size of 137 per group (health control group, pre-metabolic syndrome group and metabolic syndrome group) can achieve 80% power by using a two-sided one-way ANOVA at a significance level of 0.05.

Anthropometric and laboratory data measurements

Participants were required to conduct an overnight fasting for eight hours before the physical assessment. Anthropometric parameters such as waist circumference and body weight were determined with the standardized procedure. Then participants' standing height was measured by a stadiometer with an accuracy of 1mm under a standardized procedure. Subsequent to 5-min resting, sitting blood pressure of the right arm was determined thrice with an electronic blood pressure monitor (OMRON, HBP-1120U), the average of these readings was collected. Information, like basic demographic characteristics (age and sex), smoking status, and alcohol consumption were collected by well-trained interviewers through a computer-aided one-on-one questionnaire.

Participants were required to conduct an overnight fasting before the blood test (at least eight hours but not more than 16 hours). Approximately 5mL of venous blood samples from participants were collected, and each individual's blood sample was subsequently divided into two aliquots. One was packaged in dry ice and transported altogether to the Beijing CIC Medical Laboratory for analysis of lipid, fasting plasma glucose (FPG) and glycated hemoglobin (HbA1c). An AU5800 Chemistry Analyzer (Beckman Coulter, California, USA) was used to measure the level of total cholesterol (TC), serum low-density lipoprotein cholesterol (LDL-C), serum high-density lipoprotein cholesterol (HDL-C), serum triglycerides (TG), and FPG. Commercial reagents (Biosino, Beijing, China) were used in this measuring process. The Tosoh Automated Glycohemoglobin Analyzer HLC-723GX (Tosoh Corporation, Tokyo, Japan) was used to measure the level of glycated hemoglobin (HbA1c). The other samples were stored at -80℃ in People's hospital of Ningxia Hui Autonomous Region, and freeze-thaw cycles were strictly avoided until metabolomics analysis.

Untargeted metabolomics profiling

The pretreatment of serum specimens was as follows. 100μL of sample was taken, mixed with 400 μL of extraction solution (methanol: acetonitrile, 1:1(v/v)), the extraction solution contain deuterated internal standards, the mixed solution were vortexed for 30 s, sonicated for 10 min in 4℃ water bath, and incubated for 1 h at -40℃

to precipitate proteins. Then the samples were centrifuged at 13000 rpm (RCF=16249 (×g), R= 8.6cm) for 15 min at 4℃. The supernatant was transferred to a fresh glass vial for analysis. The quality control (QC) sample was prepared by mixing an equal aliquot of the supernatant of samples.

For polar metabolites, LC-MS/MS analyses were performed using an UHPLC system (Vanquish, Thermo Fisher Scientific) with a Waters ACQUITY UPLC BEH Amide (2.1mm×100 mm, 1.7μm) coupled to Orbitrap Exploris 120 mass spectrometer (Orbitrap MS, Thermo). The mobile phase consisted of 25 mmol/L ammonium acetate and 25 ammonia hydroxide in water（pH=9.75）(A) and acetonitrile (B). The auto-sampler temperature was 4℃, and the injection volume was 2ml (Figure S3A-B).

For non-polar metabolites, LC-MS/MS analyses were performed using an UHPLC system (Vanquish, Thermo Fisher Scientific) with a Phenomenex Kinetex C18 (2.1mm ×100 mm, 2.6 μm) coupled to Orbitrap Exploris 120 mass spectrometer (Orbitrap MS, Thermo). The mobile phase A:0.01% acetic acid in water, mobile phase B:IPA:ACN (1:1,v/v). The auto-sampler temperature was 4℃, and the injection volume was 2ml

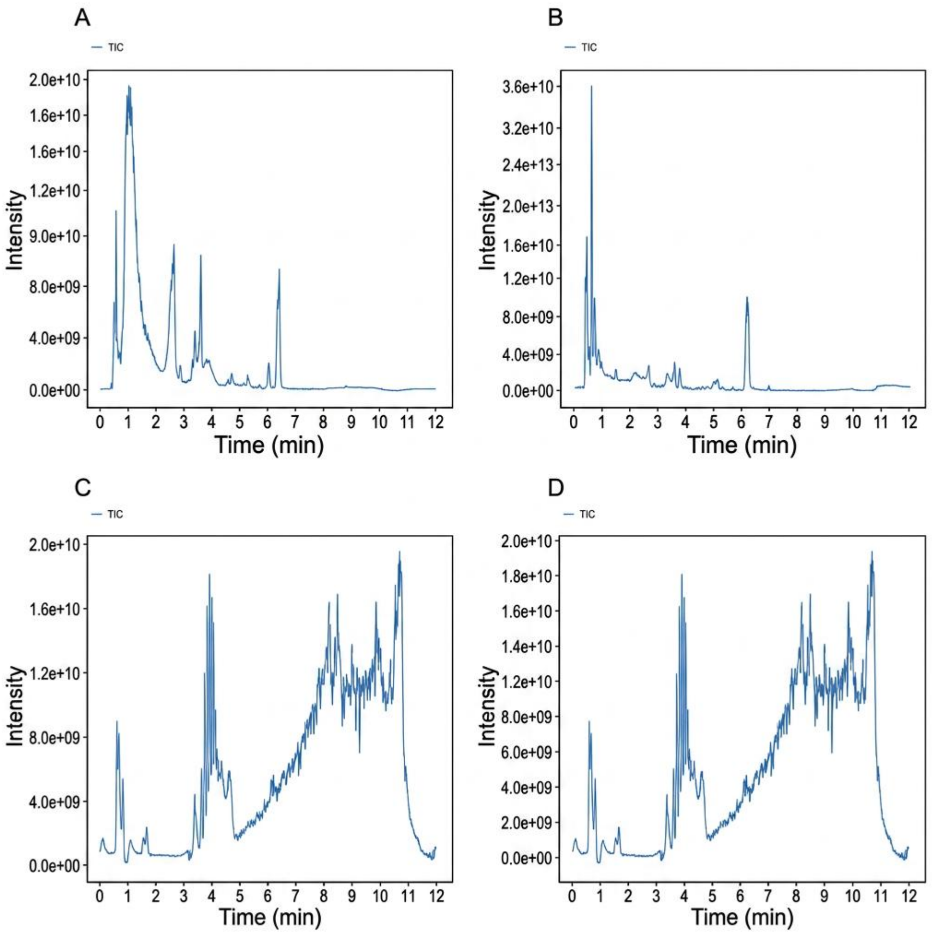

(Figure S3C-D).

Figure S3 TIC Plot of Experimental QC Samples in the untargeted metabolomic analyses. **(A)** TIC Plot of Polar Metabolite System QC Samples in Positive Ion Mode; **(B)**TIC Plot of Polar Metabolite System QC Samples in Negative Ion Mode; **(C)**TIC Plot of Non-Polar Metabolite System QC Samples in Positive Ion Mode; D: TIC Plot of Non-Polar Metabolite System QC Samples in Negative Ion Mode. TIC: Total Ion Current; QC: Quality control.

The Orbitrap Exploris 120 mass spectrometer was used for its ability to acquire MS/MS spectra on information-dependent acquisition (IDA) mode in the control of the acquisition software (Xcalibur, Thermo). In this mode, the acquisition software continuously evaluates the full scan MS spectrum. The ESI source conditions were set as following: sheath gas flow rate as 50 Arb, Aux gas flow rate as 15 Arb, capillary temperature 320℃, full MS resolution as 60000, MS/MS resolution as 15000, collision energy: SNCE 20/30/40, spray voltage as 3.8 kV (positive) or -3.4 kV (negative), respectively.

Targeted Metabolite Analysis

Calibration standard mixtures, serum samples, and internal standard solution were prepared as follows:

(1) Preparation of Calibration Standard Mixtures

Accurately weigh 10 mg of the reference standard and transfer it into a 10 mL volumetric flask. Dissolve and dilute to volume with methanol to obtain a 1 mg/mL stock solution. Dilute the stock solution with acetonitrile and combine the diluted solutions of individual reference standards to prepare a mixed standard solution with a concentration of 10 μg/mL for each analyte.

(2) Preparation of Serum Samples

Remove blood samples from the −80 °C freezer and thaw at 4 °C. Precisely pipette 50 μL of plasma into a 2 mL centrifuge tube. Add 150 μL of acetonitrile containing 10 ng/mL internal standard, vortex for 30 s, and centrifuge at 13,000 rpm for 10 min. Transfer the supernatant to a sample vial for analysis; the injection volume is 5 μL.

(3) Preparation of Internal Standard Solution

Accurately weigh 1.0 mg of the marine internal standard into a 10 mL volumetric flask. Dissolve and dilute to volume with methanol to prepare a 0.1 mg/mL stock

solution. Appropriately dilute the stock solution with acetonitrile to a final concentration of 10 ng/mL to obtain the internal standard working solution.

(4) Instrumental Analysis

Targeted metabolite analysis was performed using a TSQ Access MAX triple quadrupole liquid chromatography-tandem mass spectrometry (HPLC-MS/MS) system (Thermo Fisher Scientific, USA). Chromatographic separation was achieved on a Waters ACQUITY UPLC BEH C18 column (2.1 mm × 100 mm, 1.7 μm). The mobile phases consisted of 0.1 % formic acid in water (A) and acetonitrile (B). The column temperature was maintained at 30 °C. The injection volume was 5 μL and the flow rate was 200 μL/min. A gradient elution program was employed (see Tables S1 and S2). MS parameters were set as follows: capillary temperature 320 °C, vaporizer temperature 350 °C, sheath gas pressure 35 arb, auxiliary gas pressure 10 arb, and spray voltage ± 4.0 kV (positive) or −3.0 kV (negative); detailed settings are listed in Table S3. The system was controlled by Xcalibur software (Thermo Fisher Scientific).

Table S1 The first gradient elution conditions in the targeted metabolomics analysis

Total time (min)	A (V%)	B (V%)
0.00	95.0	5.00
1.00	95.0	5.00
2.00	60.0	40.00
3.00	20.0	80.00
4.00	5.00	95.00
5.00	95.00	5.00
6.00	95.00	5.00

Abbreviations: A is the mobile phase consisting of 0.1% formic acid in water, and B is the mobile phase consisting of acetonitrile.

Table S2 The second gradient elution conditions in the targeted metabolomics analysis

Total time (min)	A (V%)	B (V%)
0.00	97.00	3.00
2.00	97.00	3.00
4.00	80.00	20.00
5.00	60.00	40.00
7.00	20.00	80.00
8.00	5.00	95.00
9.00	97.00	3.00
10.00	97.00	3.00

Abbreviations: A is the mobile phase consisting of 0.1% formic acid in water, and B is the mobile phase consisting of acetonitrile.

Table S3 MRM conditions for 13 Metabolites in the targeted metabolomic analyses

Metabolite	Parent	Product	MRM Collision Energy	Tube lens	Polarity
4,5-Dihydro-2-methylthiazole	102.0335	42.150	25	60	+
		58.880	32	60	+
N-Methylvaline	132.1011	55.200	24	60	+
		86.150	12	60	+
Benzoyleneurea	161.0363	34.690	26	61	-
		42.190	18	61	-
1,2,2,6,6-Pentamethyl-4-piperidinol	172.1684	72.240	22	67	+
		116.130	16	67	+
(R)-3-Amino-3-(3-chlorophenyl) propionic acid	198.033	137.090	16	69	-
		162.720	20	69	-
2,6-Di-tert-butylphenol	205.1605	172.940	6	68	-
		204.650	12	68	-
4-(Chloromethyl)-7-hydroxy-8-methyl-2H-chromen-2-one	225.0331	144.410	25	67	-
		186.510	17	67	-
Procymidone	282.0093	56.550	30	97	+
		83.550	20	97	+
2-Hydroxydesmethylimipramine	283.1825	71.640	17	61	+
		206.060	50	61	+
1-Methylguanosine	298.1131	109.470	36	58	+
		134.400	37	58	+
Nonaethylene glycol	413.2395	115.840	45	66	+
		117.920	42	66	+
LPC (20:0)	552.4005	102.940	31	123	+
		551.340	59	123	+
SM(d18:1/17:0)	717.5891	182.800	23	116	+
		716.810	5	116	+

Cross-validation between untargeted and targeted metabolomics

For the eight validated metabolites, cross-platform confirmation involved three criteria: (i) retention time matching within ±0.1 min between untargeted and targeted runs; (ii) precursor ion accurate mass (<5 ppm) and fragment ion matching (Orbitrap untargeted vs. MRM transitions on triple quadrupole); (iii) authentic standard co-injection. The untargeted platform (Q-Exactive Orbitrap, full scan 70–1000 m/z) and targeted platform (triple quadrupole, MRM) were applied to independent discovery and

validation cohorts. No metabolite was included without meeting all three criteria.

Measurement and adjustment for urinary metals

The present study analyzed urine samples from 252 participants in the validation cohort for eight metal elements. The analytical approach followed the methodology established in an ongoing larger study involving 1080 participants. Concentrations of arsenic (As), cadmium (Cd), lead (Pb), manganese (Mn), copper (Cu), mercury (Hg), chromium (Cr), and zinc (Zn) were determined using inductively coupled plasma mass spectrometry (PerkinElmer NexION 1000G; PerkinElmer, USA). The instrument was operated under the following conditions: RF power 1500 W; plasma gas (argon) flow rate 15.0 L/min; auxiliary gas flow rate 1.2 L/min; nebulizer gas flow rate 0.98 L/min; sampling depth 5 mm. Data acquisition was performed with a dwell time of 20–50 ms per isotope, 20 sweeps per reading, 3 readings per replicate, and 3 replicates per sample. Helium collision cell mode was used for kinetic energy discrimination (KED), with a helium flow rate of 4.5 mL/min. Daily performance checks ensured that the oxide ratio (CeO^+ / Ce^+) was below 2.5% and the doubly charged ratio (Ba^{2+} / Ba^+) below 3.0%. Rhodium (10 μg/L) was added online as an internal standard to correct for matrix effects and signal drift.

Calibration was performed using multi-element standard solutions (PerkinElmer Pure Plus) at eight concentration levels. Blind samples and quality control samples were used throughout the process to ensure analytical reliability. For every batch of 25 test samples, a subset of urine specimens was randomly selected and processed simultaneously through microwave digestion and instrumental analysis. A relative difference of $\leq$20% between duplicate measurements was defined as the acceptance criterion. The limit of detection (LOD), the number of samples below the LOD, and their respective percentages for each metal are provided in Table S4. Metal concentrations below the LOD were imputed as LOD/ $\sqrt{2}$ in all subsequent statistical analyses.

To correct for variations in urine dilution, a covariate-adjusted standardization procedure was applied as follows: (1) a predicted creatinine level (Fit-Crea) for each participant was estimated using a regression model with ln-transformed creatinine, age, sex, weight, height, and waist circumference as covariates; (2) the ratio between the

measured ln- transformed creatinine and the predicted Fit- Crea was calculated; (3) the raw metal concentration was divided by this ratio to obtain the standardized metal level used in all statistical models.

Table S4. Distribution of urinary heavy metal concentrations (μg/L) of study population (n =1080)

Metal	LOD	< LOD *	Min	P10	P25	Mid	P75	P90	Max
As	0.011	9 (0.08)	< LOD	7.52	12.36	20.29	32.3	48.75	820.91
Cr	0.004	181 (16.76)	< LOD	2.53	6.64	15.55	32.66	93.9	204.3
Cd	0.001	160 (14.81)	< LOD	0.18	0.39	0.77	1.49	2.24	16.83
Cu	0.001	8 (0.74)	< LOD	8.06	12.98	19.58	28.42	39.2	955.80
Hg	0.002	543 (50.28)	< LOD	0.02	0.07	0.15	0.32	0.56	205.79
Mn	0.007	49 (4.54)	< LOD	2.49	5.31	10.04	16.14	24.65	178.00
Pb	0.006	444 (41.11)	< LOD	0.79	2.34	5.64	13.28	20.09	490.98
Zn	0.086	2 (0.19)	< LOD	339.69	643.98	1201.26	2211.19	3549.79	15228.31

Supplementary Results

Table S5 Comparison of baseline characteristics between the discovery and validation cohorts

Variables	Total	Discovery cohort	Validation cohort	*P* values
	（N=900）	（n=450）	（n=450）	
Gender，n(%)				0.229 [a]
Male	420 (46.47)	219 (48.67)	201 (44.67)	
Female	480 (53.33)	231 (51.33)	249 (55.33)	
Age, Median (quartile)	39 (32,48)	38 (32;46)	39 (32; 51)	<0.05 [b]
Smoking, n (%)	243 (27.00)	135 (30.00)	108 (24.00)	<0.05 [a]
Alcohol Consumption	230 (25.55)	125 (27.78)	102 (22.67)	0.078 [a]
Waist Circumference, Mean±SD	82.65±11.08	82.92±11.39	82.38±10.75	0.466 [c]
BMI, n (%)				0.985 [a]
<24.0	396 (44.00)	199 (44.22)	197 (43.78)	
24.0~27.9	298 (33.11)	149 (33.11)	149 (33.11)	
≥28.0	206 (22.89)	102 (22.67)	104 (23.11)	
Hypertension, n (%)	345 (38.33)	163 (36.22)	182 (40.44)	0.193 [a]
Hyperglycemia, n (%)	292 (32.44)	151 (33.56)	141 (31.33)	0.476 [a]
HbA1c (%), Mean±SD	5.58 ± 0.03	5.58±0.71	5.55±0.80	0.547 [c]
FPG (mmol/l), Mean±SD	5.58 ± 0.07	5.58±1.41	5.60±1.56	0.829 [c]
TG (mmol/l), Mean±SD	1.60 ± 0.80	1.60±1.71	1.46±1.24	0.166 [c]
TC (mmol/l), Mean±SD	4.21 ± 0.04	4.21±0.90	4.19±0.88	0.756 [c]
HDL-C (mmol/l), Mean±SD	1.29 ± 0.01	1.29±0.29	1.26±0.29	0.088 [c]
LDL-C (mmol/l), Mean±SD	2.51 ± 0.03	2.52±0.72	2.53±0.72	0.880 [c]

Abbreviations: MetS: Metabolic syndrome; pre-MetS: Pre-Metabolic syndrome; HC: Health Control; BMI, body mass index; HbA1c, glycated hemoglobin; FPG, fasting plasma glucose; TG, triglycerides; TC, total cholesterol; HDL-C, high-density lipoprotein cholesterol; LDL-C, low-density lipoprotein cholesterol.

[a] *P* values were obtained from chi-square test.

[b] *P* value was obtained from Kruskal–Wallis rank-sum test.

[c] *P* values were obtained from ttest.

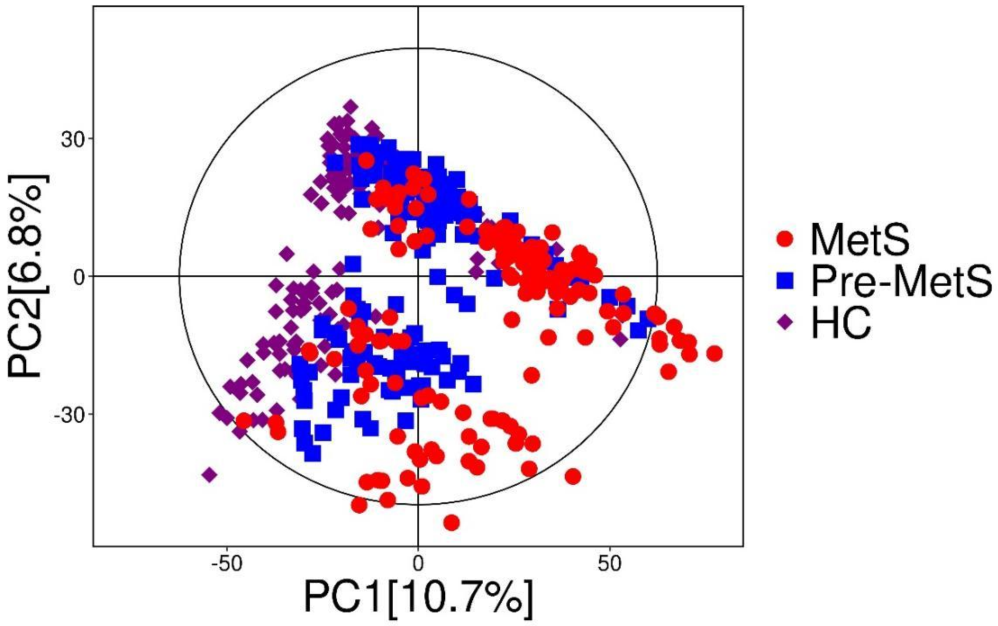

Figure S4 The total PCA Score Plot of the Three Groups in the untargeted metabolomic analyses. Colors and shapes display the subjects from different groups. PCA: Principal Component Analysis.

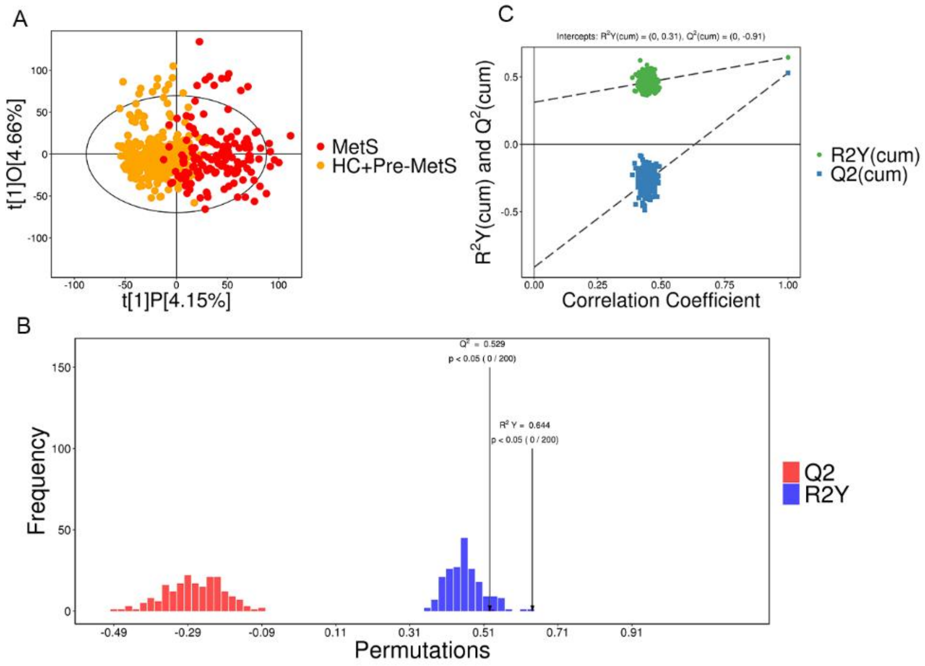

Figure S5 Metabolic profiling of serum samples from patients with Mets and pre-MetS (+) HC in the untargeted metabolomic analyses. (A) The OPLS–DA score plot based on the combinational data of ESI+ and ESI− modes. (B) OPLS-DA Permutation Plot Comparing HC (+) pre-MetS and MetS (C) Validation of the OPLS–DA model by the permutation test.

Colors and shapes display the subjects from different groups. MetS: Metabolic syndrome; pre-MetS: Pre- Metabolic syndrome; HC: Health Control; PCA: principal component analysis; OPLS–DA: orthogonal projections to latent structures discriminate analysis.

Table S6. CV- ANOVA results for the OPLS- DA model [a]

Source	SS	DF	MS	F	P	SD
Total corr.	299.00	299	1.00	-	-	1.00
Regression	212.18	4	53.04	180.23	<0.0001	7.28
Residual	86.82	295	0.29	-		0.54

Abbreviations: SS, sum of squares; DF, degrees of freedom; MS, mean square; F, F– statistic; SD, standard deviation.

[a] Comparison between healthy controls (HC) and the combined group of pre- metabolic syndrome (Pre- MetS) and metabolic syndrome (MetS) patients.

Table S7 208 Candidate Metabolomic Biomarkers of MetS in the untargeted metabolomic analyses

Metabolites	RT (min)	m/z	FC	VIP	*P* value
Phytanic acid	34.1	311.2957	0.588942758	2.278423618	7.71995E-18
Indolelactic acid	141.5	204.0667	1.566915014	2.634187419	4.7698E-12
alpha-Tocopherol (Vitamin E)	29.7	429.3735	0.426394003	2.074417849	4.81607E-27
Terephthalic acid	358.4	165.0195	0.950256668	2.284946264	4.50367E-08
Glucose	304.4	179.0562	1.271099778	2.055900407	3.13199E-11
beta-Alanine	354.4	88.0403	1.224032709	2.21590141	5.33487E-13
2-Ketocaproic acid	35.4	129.0556	1.220805247	2.04181818	2.75437E-10
1,5-Anhydroglucitol	200	163.0613	0.471889235	3.269872266	4.05859E-31
Citrulline	42.4	174.0891	0.813003691	2.096187645	2.44209E-12
Acetylproline	95.6	156.0674	1.42891583	2.060333102	5.23103E-15
Alanine	354.4	88.0403	1.246603507	2.369308899	6.69252E-15
Tryptophan	275.4	205.0961	1.323957842	2.590045562	3.28857E-14
Formylmethionine	201.6	176.0388	1.236511426	2.471693171	5.21473E-11
Benzoyleneurea	252.5	161.0363	0.95381227	2.164331062	2.28088E-08
LPC(20:0)	210.1	552.4005	0.64139848	2.366397949	1.05893E-23
1-Methylguanosine	204.8	298.1131	1.492660009	2.468949291	1.07569E-07
PC(16:0/18:0)	159	744.5859	0.454686754	3.091502738	7.94549E-32
Nonaethylene glycol	68.5	413.2395	1.2502353	2.049790955	5.80229E-09
PC(17:0/17:0)	159	744.5859	0.452521713	3.096524751	2.64409E-32
PC(18:0/16:0)	159	744.5859	0.454241933	3.092189732	1.82342E-32
Tyrosine	313.4	182.0803	1.48493371	2.382771687	5.74055E-16
PE(P-38:6)	146.8	748.5254	0.45924943	2.837358554	3.13954E-25
PC(34:3)	146.7	738.5382	0.343099413	2.114486688	3.80911E-18

Table S7 Continued

Metabolites	RT (min)	m/z	FC	VIP	*P* value
p-Dimethylaminobenzaldehyde	337.7	150.0904	0.931157148	2.237463998	6.82759E-14
Hexadecatetraenoylcarnitine	202.3	392.2776	0.732181153	2.076882547	8.69453E-11
SM(d18:1/17:0)	201.6	717.5891	0.438284902	2.173991868	2.57254E-24
Palmitoyl sphingomyelin	202.5	703.5699	0.503518932	2.914727133	2.55976E-29
Arjunolic acid	87.1	487.3423	1.329740476	2.023341236	1.46481E-06
2,4-Dihydroxybutanoic acid	305.3	101.0244	1.273194384	2.397469666	1.94645E-11
SM(d18:1/18:0)	184.8	731.6012	0.553566365	2.453846545	1.49019E-20
Palmitoylcarnitine (Car(16:0))	183.9	400.3405	1.325499224	2.372729588	4.68529E-16
4-Oxohexanoic acid	214	129.0561	1.27494424	2.341296999	3.58196E-10
4-Chlorophenylalanine	255.8	200.0464	1.134633354	2.290295922	2.23011E-08
SM(d36:2)	200.9	729.5862	0.507084782	2.795572965	2.46063E-21
Oxotetradecenoylcarnitine	200.6	384.2707	0.472830057	3.129293704	2.47829E-25
PC(42:5)	147.4	846.6323	0.341935559	2.930870053	1.57283E-38
PC(40:5)	148.8	818.6027	0.496251778	2.661702507	1.89013E-29
Sarcosine	354.4	88.0403	1.224032709	2.21590141	5.33487E-13
Tetracosanoic acid	599.7	367.3591	0.927960877	2.077062133	1.50372E-09
2-Methylguanosine	204.8	298.1131	1.466851273	2.47531923	1.04213E-07
o-Tyrosine	313.4	182.0803	1.48493371	2.382771687	5.74055E-16
Carbidopa	71.5	227.1026	1.412074621	2.331015962	1.6095E-14
7-(4-Methoxyphenyl)-2,4-pteridinediol	296.3	269.0678	0.937913148	2.44215514	2.97032E-15
Glabrol	233.8	393.2069	0.958053718	2.119960511	2.79956E-06
Hydroxyhydroquinone	306.5	125.0244	1.234476083	2.212650866	8.48624E-10
(R)-3-Amino-4-methylpentanoic acid	276.6	130.0873	1.219223476	2.467309829	3.83141E-12

Table S7 Continued

Metabolites	RT (min)	m/z	FC	VIP	*P* value
1-Isopropyl-1H-benzimidazole-5-carboxylic acid	275.6	203.0827	1.348631293	2.58767507	4.96884E-15
Desethylatrazine	275.4	188.0697	1.331234495	2.594457348	2.93195E-14
Procymidone	129.4	282.0093	1.266682491	2.024259887	4.52312E-16
L-Ribulose	304.5	149.0456	1.260885769	2.073181753	1.10335E-10
1-Chloro-3-(triphenylphosphoranylidene)acetone	285.2	353.0856	1.21592834	2.020334454	9.99752E-08
6-Methoxy-1,7-bis(3,4-dihydroxyphenyl)heptan-3-one	275.9	359.1503	1.33354939	2.154137978	4.90436E-09
3,5-Dihydroxydecanoic acid	194.4	203.129	0.539887876	2.024384806	0.000252058
PC(42:11)	209.7	852.5549	0.378530939	2.437359814	3.15699E-27
Muramic acid	209.1	252.1074	0.914244479	2.494284965	2.42415E-10
8-Hydroxy-2'-deoxyguanosine	332.1	282.0847	1.4930429	2.615050406	3.83794E-15
Nelarabine	316.2	296.1003	1.468728838	2.575488587	9.0833E-13
3-Aminopentanoic acid	309.7	116.0716	1.20074072	2.246001715	1.628E-10
(2-([3-hydroxy-2-tetradecanamidooctadec-4-en-1-yl phosphonato]oxy)ethyl)trimethylazanium	204.1	733.5493	0.683382473	2.116125234	3.05876E-16
(S)-3,4-Dihydroxybutyric acid (lithium hydrate)	300.9	119.0349	1.209518614	2.132869741	4.7394E-11
Sulfadimidine	389.9	279.0917	0.945182313	2.364318031	1.47113E-12
1,3-Dihydroxyacetone dimer	283.7	179.0563	1.277202005	2.413277192	1.60819E-13
Heptane-1-thiol	276.1	133.1048	1.201447321	2.299306836	1.46435E-09
Dodecylbenzenesulfonic acid	581.9	325.1853	0.942136363	2.139193016	1.31823E-12
2,6-Di-tert-butylphenol	495.5	205.1605	0.933346851	2.861176995	9.29154E-17

Table S7 Continued

Metabolites	RT (min)	m/z	FC	VIP	*P* value
Piperidine	276.5	86.0961	1.190695263	2.209914181	5.0773E-09
Isovaline	309.3	118.0859	1.146781522	2.048476632	7.3511E-08
(R)-3-Amino-3-(3-chlorophenyl)propionic acid	256	198.033	1.100591654	2.230261332	3.58123E-09
[3-(Hexadecanoyloxy)-2-[icosa-5,8,11-trienoyloxy]propoxy]({[2,3,4,5,6-pentahydroxycyclohexyl]oxy})phosphinic acid	206.9	859.5315	0.329064245	2.995490515	2.59671E-35
Amidosulfonic acid	656.6	97.9909	0.943013283	2.636713173	1.41692E-12
4-Fluoro-N-[3-(trifluoromethyl)phenyl]benzamide	275.5	284.0694	1.237399334	2.298612897	1.40719E-10
Chatenaytrienin 3	184.3	541.5	0.301467137	2.537203951	9.48178E-25
1-(3,4-Dihydroxyphenyl)-2-(2-ethyl-1H-imidazol-1-yl)ethan-1-one	290.5	245.0942	1.333234028	2.177563735	3.94832E-12
19-Noretiocholanolone glucuronide	360.6	451.2316	2.568851328	2.293566508	2.33151E-15
Isopongaflavone	275.2	335.1262	1.275072031	2.291457199	2.71201E-08
Trifolirhizin	355.8	469.1075	0.934648734	2.730327968	1.72647E-16
(3-Carboxypropyl)trimethylammonium cation	389.5	146.1169	0.814210497	2.114770883	5.71571E-10
1-[3,5-Di-tert-butyl-4-hydroxyphenyl]-2-[2-[(3-hydroxypropyl)amino]-5,6-dimethyl-1H-benzimidazol-1-yl]ethan-1-one	200.4	466.3041	0.331394047	3.152356636	1.16101E-27
1-(1Z-Octadecenyl)-2-(5Z,8Z,11Z,14Z-eicosatetraenoyl)-sn-glycero-3-phosphocholine	52.3	794.6019	0.500460435	2.580274004	8.20284E-24
2-Cyano-L-phenylalanine	38.3	191.0805	0.74395611	2.186393721	2.23513E-10
2-Methylbutylamine	647.8	88.1117	0.94700927	2.269917832	3.3234E-10

Table S7 Continued

Metabolites	RT (min)	m/z	FC	VIP	*P* value
N-Methylvaline	66.5	132.1011	1.189510395	2.218615046	1.62601E-13
4-Amino-6-chloropyrimidine-5-carbaldehyde	202.7	158.0119	0.815333207	2.712330564	1.07908E-17
Ubiquinol 8	201	731.6009	0.461693239	2.767566423	4.72925E-23
1-O-Hexadecyl-2-O-(4Z,7Z,10Z,13Z,16Z,19Z-docosahexaenoyl)-sn-glyceryl-3-phosphorylcholine	51.5	792.5859	0.560526898	2.102266756	9.48569E-17
2,3,4,5-Tetrahydro-6-(5-methyl-2-furanyl)pyridine	324.3	164.106	0.936791525	2.471824775	8.37769E-13
Soyacerebroside II	202.4	714.5563	0.342360153	2.61958974	2.616E-32
Montecristin	209.6	575.5003	0.254248334	2.27181388	3.04903E-24
Aureonitol	120.8	207.1391	1.208434697	2.513244941	1.27549E-14
Irisflorentin	200.4	387.1103	0.446014484	2.87216385	7.93785E-31
1-Hexadecyl-2-(9Z-octadecenoyl)-sn-glycero-3-phosphocholine	159.3	746.6005	0.393059204	3.208295827	5.33293E-37
1-(1Z-Octadecenyl)-2-(5Z,8Z,11Z,14Z-eicosatetraenoyl)-sn-glycero-3-phosphoethanolamine	145.9	752.5548	0.309846625	2.986310359	4.17209E-30
N-(3-Acetylphenyl)-4-fluorobenzamide	385.7	258.0932	0.950505378	2.026141978	1.16527E-07
1-(9H-Purin-6-yl)piperidin-4-amine	307.2	219.1367	0.926168157	2.542283183	3.97487E-20
Austdiol	288.9	237.0744	0.945128088	2.043245203	6.32367E-08
6-((5-Fluoro-2-((3,4,5-trimethoxyphenyl)amino)pyrimidin-4-yl)amino)-2,2-dimethyl-2H-pyrido[3,2-b][1,4]oxazin-3(4H)-one	256.9	471.1746	0.389105514	2.304357035	7.57426E-19

Table S7 Continued

Metabolites	RT (min)	m/z	FC	VIP	*P* value
LPC(32:0)	163.1	720.5864	0.346844273	2.682320653	1.22057E-31
2-Hydroxy-6-methyl-4-(trifluoromethyl)nicotinonitrile	654.1	201.0266	0.94951483	2.853830426	1.63235E-13
5-Methyl-2'-deoxycytidine	323.2	240.1	0.945463077	2.145490602	5.21105E-10
1,3,5-Trimethyl-2,4,6-tris(3,5-di-tert-butyl-4-hydroxybenzyl)benzene	201.1	773.5812	0.409594324	2.777117833	2.03837E-21
N-(2,6-Dimethylphenyl)benzamide	402.5	226.1211	0.933385186	2.218835805	1.6892E-10
4,4,4-Trifluorobutyric acid	37.1	141.0173	0.955299924	2.42763927	2.13499E-10
1-(beta-D-Ribofuranosyl)-1,4-dihydronicotinamide	163.4	257.1119	1.241148373	2.187149409	4.26266E-07
PC(35:5)	54.1	748.5196	0.67556196	2.116636777	7.02913E-15
1-O-Hexadecyl-2-O-(2E-butenoyl)-sn-glyceryl-3-phosphocholine	543.4	550.3843	0.773705512	2.292185189	5.51062E-16
6-Chloro-1-phenyl-2,3,4,5-tetrahydro-1H-3-benzazepine-7,8-diol	283.6	288.0765	1.160667346	2.046545114	4.78202E-07
3-Chloro-2-hydroxy-5-phenylbenzoic acid	657.5	247.0148	0.958692252	2.010579009	2.01849E-06
Kaempferol 3-O-xyloside	370.8	417.0845	0.948168317	2.646469173	3.24338E-12
2-Anilinocyclohexanone	363.7	190.1248	0.944217701	2.356500395	5.72623E-11
Thieno[3,2-b][1]benzothiophene-2-carboxylic acid	2.6	232.9764	0.946164847	2.106002198	1.00598E-10
[4-(4-Hydroxy-2-quinazolinyl)phenyl]methanesulfonamide	330.5	314.057	1.352350411	2.011145594	8.07988E-11

Table S7 Continued

Metabolites	RT (min)	m/z	FC	VIP	*P* value
7-Hydroxy-6-methoxy-alpha-pyrufuran	288.7	321.0929	0.928150797	2.188942101	7.4875E-13
1,3-Bis[3-(4,5-dihydro-1H-imidazol-2-yl)phenyl]urea	227.8	349.1807	0.953090897	2.163295026	2.51434E-05
2-Aminopyridine-5-carbothioamide	255.7	154.0413	1.133094298	2.231083027	1.73952E-07
Methyl 2-{[(4-methoxyphenyl)carbonyl]amino}-5,6-dihydro-4H-cyclopenta[b]thiophene-3-carboxylate	310.9	330.085	1.401275964	2.267140031	2.18808E-13
1,4-Piperazinediethanesulfonic acid	275.5	303.0637	1.231096355	2.442205139	6.65511E-12
(2-Aminoethoxy)[2-[docosa-4,7,10,13,16,19-hexaenoyloxy]-3-[hexadec-1-en-1-yloxy]propoxy]phosphinic acid	143.9	746.5132	0.417584385	2.890768903	5.13074E-27
PC(22:4(7Z,10Z,13Z,16Z)/P-18:0)	149.4	822.6338	0.385265003	2.681682684	5.86927E-35
3-cis-Hydroxy-beta,epsilon-caroten-3'-one	30.2	551.4203	0.286989377	2.928236479	2.98365E-32
Methyl 2-(4-chlorophenyl)acetate	43.4	185.0407	1.913790298	2.534990925	3.52831E-09
5-Chloro-2,4-difluorobenzoic acid	2.6	190.9658	0.94799913	2.039807725	1.84174E-09
Flufenacet ethanesulfonic acid	309.5	274.0611	1.148756494	2.250591772	5.81708E-09
all-trans-Decaprenyl diphosphate	198.1	859.5731	0.210775109	3.391784713	5.48894E-30
PC(22:6(4Z,7Z,10Z,13Z,16Z,19Z)/P-18:1(11Z))	145.6	816.5867	0.535204261	2.544927952	9.7043E-27
2-Amino-3-({[3-(hexadecanoyloxy)-2-(octadecanoyloxy)propoxy](hydroxy)phosphoryl}oxy)propanoic acid	86.6	762.5081	0.474281627	2.039824987	4.70675E-10
PC(22:4(7Z,10Z,13Z,16Z)/P-18:1(9Z))	49.5	820.618	0.394345281	2.559939134	5.19919E-25
Cuminyl alcohol	66.4	133.1066	0.94141183	2.324650901	8.50178E-09

Table S7 Continued

Metabolites	RT (min)	m/z	FC	VIP	*P* value
N-Demethylmifepristone	275.4	416.2596	1.558493091	2.073110258	1.42301E-09
Tafluprost	360.8	453.2431	2.680868348	2.164672141	4.72714E-14
17-Methylene-4-androsten-3-one	351.9	285.216	0.928588524	2.173398657	2.80245E-08
PC(22:5(7Z,10Z,13Z,16Z,19Z)/22:6(4Z,7Z,10Z,13Z,16Z,19Z))	208.2	880.5877	0.265588706	3.09538608	1.31065E-32
Hexacosanoylcarnitine	184.3	540.4966	0.334332401	2.467364102	1.80957E-24
5-(2-Thienylmethylene)-2,4,6(1H,3H,5H)-pyrimidinetrione	656.1	220.997	0.944068676	2.819030404	1.02182E-13
1-Stearoyl-2-linoleoyl-sn-glycero-3-phospho-(1'-rac-glycerol)	35.6	773.5337	0.246049549	2.699133105	8.00155E-25
Cryptocapsone	29.8	567.4147	0.460587156	2.673366121	8.10748E-27
PC(20:2(11Z,14Z)/20:5(5Z,8Z,11Z,14Z,17Z))	150.7	832.5809	0.650040429	2.509767514	1.12354E-23
N-Isobutyl-3-methylbutanamide	395.3	158.1529	0.952307606	2.49334824	7.73902E-11
1-(3,4-Dihydroxyphenyl)-6,7-dihydroxy-1,2-dihydronaphthalene-2,3-dicarboxylic acid	371.2	357.0628	0.95596703	2.45250823	4.267E-12
PI(18:1(9Z)/20:4(5Z,8Z,11Z,14Z))	208.2	885.5441	0.222921678	2.542915971	7.81717E-16
PC(20:2(11Z,14Z)/15:0)	159.6	772.5813	0.580477049	2.322506801	3.2838E-17
PG(18:0/18:0)	145.5	779.5764	0.147218545	3.023268581	8.21021E-33
N-(1H-Indol-3-ylacetyl)alanine	291.4	247.1064	1.429579846	2.248582111	1.76176E-10
PC(20:4(8Z,11Z,14Z,17Z)/20:1(11Z))	151.7	836.6101	0.533174029	2.577391869	4.58301E-25
Xanthophyll	30.3	568.4234	0.332478427	2.905702731	1.35185E-36
PG(16:1(9Z)/18:0)	146.7	749.528	0.47259038	2.767282227	1.31576E-23

Table S7 Continued

Metabolites	RT (min)	m/z	FC	VIP	*P* value
1-Phenyl-2,3,4,5-tetrahydro-1H-3-benzazepine	120.6	224.1433	1.22473401	2.699598286	6.82378E-14
(2-Aminoethoxy)[3-[hexadec-1-en-1-yloxy]-2-[icosa-5,8,11,14-tetraenoyloxy]propoxy]phosphinic acid	145.7	722.5135	0.390114774	2.769871526	1.29352E-25
3,7,8-Trihydroxy-3-methyl-10-oxo-1,4-dihydropyrano[4,3-b]chromene-9-carboxylic acid	196.6	307.0517	0.711621948	2.283419078	2.61384E-11
3-(Trifluoromethyl)cinnamic acid	283.1	215.0332	1.318958069	2.226623625	4.35589E-10
PC(20:3(5Z,8Z,11Z)/18:0)	154.2	812.6089	0.521096789	2.565961402	7.77655E-27
beta-Cryptoxanthin	29.6	552.4278	0.216699175	3.068623964	2.33432E-27
N-(3-Acetylphenyl)ethanesulfonamide	199.8	226.0568	0.569160853	2.767874191	3.11476E-27
(2-Aminoethoxy)[2-[docosa-4,7,10,13,16,19-hexaenoyloxy]-3-[octadeca-1,9-dien-1-yloxy]propoxy]phosphinic acid	140.5	772.5279	0.386012888	2.505910034	4.43434E-28
SM(d16:1/24:1(15Z))	198	785.6511	0.282960467	3.221818768	8.80914E-29
PC(18:2(9Z,12Z)/P-16:0)	155.6	742.5706	0.468403353	3.020609974	3.87168E-39
N-(3-Methyl-1-phenyl-1H-pyrazol-5-yl)-N'-phenylurea	87.4	293.1416	1.432901031	2.565484782	4.34148E-17
(2-Aminoethoxy)[2-[docosa-4,7,10,13,16,19-hexaenoyloxy]-3-[octadec-1-en-1-yloxy]propoxy]phosphinic acid	86.9	774.5424	0.173286868	3.25944149	4.14136E-25

Table S7 Continued

Metabolites	RT (min)	m/z	FC	VIP	*P* value
SM(d18:1/20:0)	199.4	759.6341	0.368462199	3.050560439	9.50383E-28
[2-[Docosa-4,7,10,13,16,19-hexaenoyloxy]-3-(octad pentahydroxycyclohexyl]oxy})phosphinic acid	203.3	909.5519	0.271910976	2.739710722	5.953E-28
SM(d17:1/24:1(15Z))	197.5	799.6665	0.270860654	3.262200744	8.74298E-27
PC(20:0/18:2(9Z,12Z))	155.5	814.6213	0.439340802	2.749409555	1.12137E-23
(4-((1H-Imidazol-2-yl)methyl)piperidin-1-yl)(4'-fluoro-[1,1'-biphenyl]-4-yl)methanone	275.9	362.1688	1.135927593	2.113000835	5.82296E-07
3-Oxocyclobutanecarboxylic acid	304.6	113.0243	1.241655278	2.065943453	4.44133E-10
(3beta,5alpha,6beta,22E,24R)-23-Methylergosta-7,22-diene-3,5,6-triol	29.3	445.3654	0.45669144	2.95156189	1.4181E-34
beta.-Hydroxyphenylalanine	313.1	180.0668	1.422866119	2.345349594	2.45096E-14
PE(18:3(9Z,12Z,15Z)/P-18:1(9Z))	148.7	724.5256	0.42601429	2.535273366	3.0715E-25
Ergostane-3,6-dione	29.2	415.3544	0.475562782	2.410627439	7.95854E-23
Talinolol	203.4	364.2553	0.348403666	3.080443646	1.95951E-22
SM(d18:1/22:0)	198.4	787.6633	0.316136369	2.879948395	2.68836E-27
(2-Aminoethoxy)[2-[icosa-5.8.11.14-tetraenoyloxy]-3-[octadec-11-enoyloxy]propoxy]phosphinic acid	86.7	764.5215	0.312085015	2.528631893	6.25359E-18
Lazabemide	656.5	200.0582	0.947772414	2.11706622	1.2434E-08
PI(16:1(9Z)/18:1(9Z))	209.7	835.53	0.42552414	2.090705264	9.13397E-23

Table S7 Continued

Metabolites	RT (min)	m/z	FC	VIP	*P* value
Mangiferdesmethylursanone	29.4	429.3699	0.473401981	2.549090621	1.10831E-30
2-Bromo-5-fluoro-N-(2-furylmethyl)benzamide	655.9	297.9894	0.941013792	2.415444101	4.26032E-11
Frangulin B	362.4	401.0889	0.950503098	2.602786532	2.26678E-13
(3beta,5xi,9xi,13xi,16beta,17xi,23S)-20-Hydroxy-16,23:16,30-diepoxydammar-24-en-3-yl 6-deoxy-alpha-L-mannopyranosyl-(1->2)-[beta-D-xylopyranosyl-(1->2)-beta-D-glucopyranosyl-(1->3)]-alpha-L-arabinopyranoside	204.1	1043.5371	0.193937831	3.14872315	2.98324E-27
SM(d17:1/24:0)	197.8	801.679	0.236018457	2.813349709	1.84807E-23
1-Stearoyl-2-oleoyl-sn-glycero-3-phospho-(1'-sn-glycerol)	210.3	775.5593	0.517348449	2.647203574	3.93598E-32
Puerariaglycoside 3	375.9	445.1154	0.934466375	2.402859476	7.606E-12
Pyridine	54.6	80.049	0.913865283	2.482911282	8.59547E-17
Antibiotic SB_202742	202.7	371.2624	0.554252868	2.83314049	1.56242E-24
4'-O-beta-D-Glucosyl-5-O-methylvisamminol	309.8	453.1738	1.274976886	2.200874951	5.56681E-07
(R)-4-((1R,3S,5S,7R,8S,9S,10S,13R,14S,17R)-1,3,7-trihydroxy-10,13-dimethylhexadecahydro-1H-cyclopenta[a]phenanthren-17-yl)pentanoic acid	199.3	431.2724	0.534322811	2.169170865	1.05617E-13
Benzophenone-4	122.8	307.0298	2.092059283	2.816691879	8.14695E-17
M131T305	304.7	131.035	1.27946078	2.320525246	2.67602E-12
M386T68	68.4	386.0129	1.412836151	2.042254675	4.9386E-08

Table S7 Continued

Metabolites	RT (min)	m/z	FC	VIP	*P* value
Wogonoside	366.2	459.0955	0.943830844	2.366910526	5.4559E-13
(2-Aminoethoxy)[2-[octadec-9-enoyloxy]-3-(octadecanoyloxy)propoxy]phosphinic acid	157.9	744.5488	0.537169856	2.156221492	1.93925E-19
PE(20:0/20:3(5Z,8Z,11Z))	156.8	798.5952	0.569342916	2.464460491	3.4783E-20
N-Butyl-2-(2,4-dichlorophenoxy)propanamide	304.2	290.0745	2.115603011	2.10166921	0.000231856
PE(20:4(8Z,11Z,14Z,17Z)/P-18:1(9Z))	145.6	750.5393	0.381642012	3.05101643	1.28213E-31
beta.-Estradiol 17-valerate	204.2	357.2471	0.530685853	2.361908437	6.77585E-16
PE(18:2(9Z,12Z)/P-18:1(9Z))	151.2	726.5384	0.36414612	2.986851163	5.24721E-30
PS(22:0/22:0)	144.7	904.7114	0.113821705	2.839714524	2.83671E-24
Peonidin 3-sambubioside 5-glucoside	27.4	758.2167	0.692759869	2.656227281	4.56584E-19
2-[Bis(5-methyl-2-furyl)methyl]-6-bromo-4-nitrophenol	35.8	390.0016	0.946364024	2.197619177	1.47873E-11
Neoacrimarine G	309.3	516.158	1.202872635	2.101187648	7.3091E-07
Dopamine 4-beta-D-glucuronide	298.3	328.1074	1.431820702	2.523350126	5.90593E-12
4-Bromo-5-fluoro-2-methylbenzonitrile	314.8	213.9687	0.837487817	2.619933988	1.19467E-11
Tyrosylphenylalanine	199.5	327.1298	0.204943201	3.113753502	5.97142E-22
(+)-Epicubenol	379	240.2307	0.954138274	2.080995404	5.00146E-08
M239T199	199.3	238.9983	0.426720464	3.171010419	2.53185E-31
M337T259	259.1	337.058	0.930245714	2.260120232	5.64679E-13
N-Succinyl-LL-2,6-diaminoheptanedioate	329.9	308.1451	1.564996808	2.227958916	3.77489E-16
M424T477	477.1	424.2202	0.94066447	2.1251126	9.86111E-08
3-Ketosucrose	275.6	361.0721	1.249240296	2.484878442	6.83828E-13
1-Methoxy-2-hydroxyanthracene	200	263.0426	0.403575107	3.012197316	7.27897E-30

Abbreviations: RT, retention time; m/z, mass-to-charge ratio; FC, fold change; VIP, variable importance in projection.

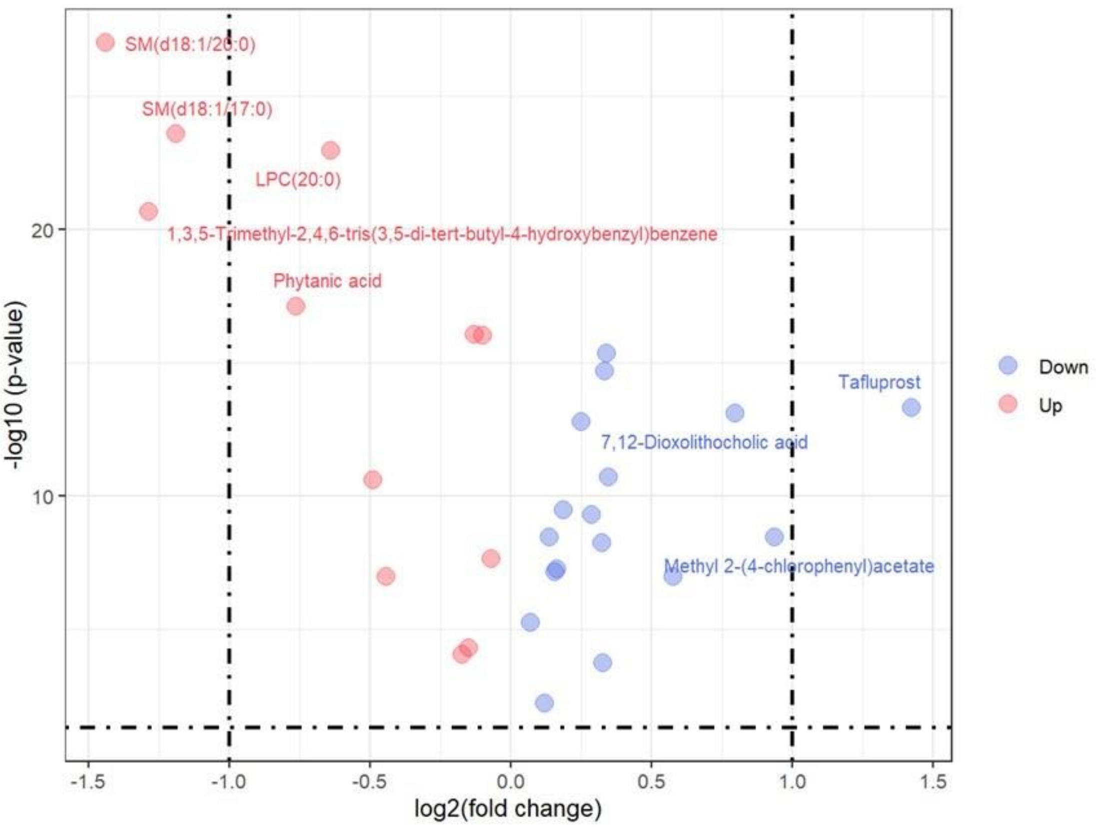

Figure S6 The volcano plot depicts the difference of metabolites between MetS and non-MetS patients in the untargeted metabolomics analysis. A dot represents a metabolite.

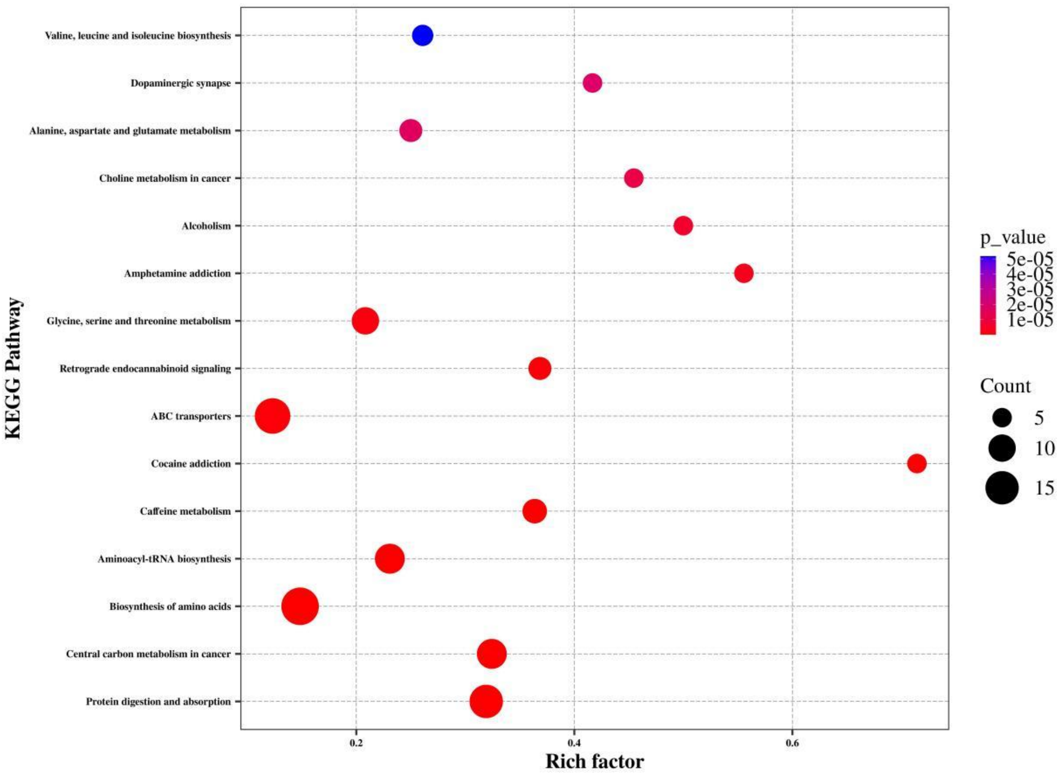

Figure S7 KEGG pathway enrichment analysis of the 208 differential metabolites. Each bubble represents a pathway. The x-axis shows the rich factor (proportion of mapped metabolites in the pathway). The bubble size is proportional to the pathway impact value. Colours indicate $-\log_{10}$ (p-value), with red representing higher significance.

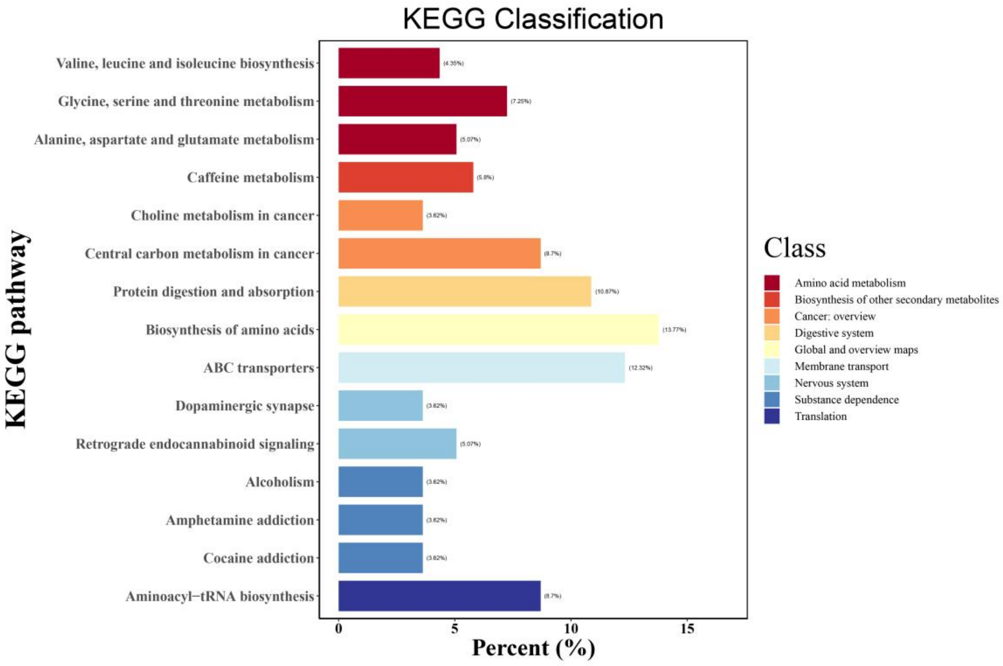

Figure S8 Classification of enriched KEGG pathways. The bars show the percentage of mapped metabolites within each top-level KEGG category (amino acid metabolism, nervous system, substance dependence, etc.).

Table S8. Identification parameters of the 29 high- confidence candidate metabolites detected by untargeted metabolomics.

Metabolite	RT (min)	Precursor m/z	Ion mode	MS/MS fragment ions (m/z) [a]	Database (source, score) [b]	MSI level [c]	Reference (HMDB ID / PMID) [d]
Benzoyleneurea	252.5	161.0363	[M-H]-	161.03, 115.92, 102.93, 73.03	NGM_indatabase (3.88)	1	HMDB0249051
1-Methylguanosine	204.8	298.1131	[M+H]+	298.11, 166.07, 140.00, 98.98	NGM_indatabase (3.88)	1	HMDB0001563
Nonaethylene glycol	68.5	413.2395	[M-H]-	413.24, 325.18, 281.16, 237.13, 193.11	NGM_indatabase (3.88)	1	-
2,4-Dihydroxybutanoic acid	366.7	152.07	[M+H]+	101.02, 83.01, 73.03, 59.01, 57.03	NGM_indatabase (3.82)	1	HMDB0000360
Indole-3-pyruvic acid	57.5	202.051	[M-H]-	202.05, 174.06, 158.06, 130.07, 128.05	NGM_indatabase (3.80)	1	HMDB0060484
S-Methylmethionine	74.2	147.0758	[M-H2O+H]+	102.06, 147.08, 88.04, 87.05, 60.04	NGM_indatabase (3.49)	1	HMDB0038670
(R)-3-Amino-3-(3-chlorophenyl)propionic acid	256.0	198.033	[M-H]-	198.03, 181.01, 162.06, 137.02, 125.02	NGM_pbdatabase (2.74)	2	HMDB0243595
N-Methylvaline	66.5	132.1011	[M+H]+	132.10, 86.10, 87.10, 69.07	NGM_pbdatabase (2.65)	2	HMDB0061716
1,2,2,6,6-Pentamethyl-4-piperidinol	431.5	172.1684	[M+H]+	172.17, 109.10, 102.09, 88.08, 131.09	NGM_pbdatabase (2.64)	2	HMDB0036195
4-(Chloromethyl)-7-hydroxy-8-methyl-2H-chromen-2-one	40.8	225.0331	[M+H]+	225.04, 209.03, 164.03, 158.98, 135.00	NGM_pbdatabase (2.58)	2	-
2-Imino-1-imidazolidineacetic acid	280.5	142.0611	[M-H]-	142.06, 98.07, 99.08, 60.02, 59.01	NGM_pbdatabase (2.47)	2	HMDB0250642

Table S8 Continued

Metabolite	RT (min)	Precursor m/z	Ion mode	MS/MS fragment ions (m/z) [a]	Database (source, score) [b]	MSI level [c]	Reference (HMDB ID / PMID) [d]
2-Hydroxydesmethylimipramine	29.8	283.1825	[M+H]+	266.18, 226.88, 201.06, 154.05, 119.09	NGM_pbdatabase (2.46)	2	HMDB0060992
Oxacillin	209.3	420.0596	[M+H]+	198.03, 200.03, 181.01, 199.04, 162.06	NGM_pbdatabase (2.22)	2	HMDB0014851
4,5-Dihydro-2-methylthiazole	205.6	102.0335	[M+H]+	79.02, 81.52, 70.01, 56.96, 74.10	NGM_pbdatabase (2.16)	2	HMDB0029555
SM(d18:1/17:0)	201.6	717.5891	[M+H]+	184.07, 717.58, 104.11, 86.10, 708.41	NGM_indatabase (3.84)	1	HMDB0240609
1,3,5-Trimethyl-2,4,6-tris(3,5-di-tert-butyl-4-hydroxybenzyl) benzene	201.1	773.5812	[M-H]-	773.59, 713.56, 699.54, 714.58, 700.54	NGM_pbdatabase (2.58)	2	-
3,7,8-Trihydroxy-3-methyl-10-oxo-1,4-dihydropyrano[4,3-b]chromene-9-carboxylic acid	196.6	307.0517	[M-H]-	307.05, 263.20, 289.18, 137.04, 226.80	NGM_pbdatabase (2.37)	2	HMDB0252514
SM(d18:1/20:0)	199.4	759.6341	[M+H]+	184.07, 759.65, 104.11, 86.10, 124.99	NGM_pbdatabase (2.30)	2	HMDB0012102
Biliverdin	426.0	581.2435	[M-H]-	285.12, 497.28, 241.13, 286.13, 239.12	NGM_indatabase (3.88)	1	HMDB0001008
2,6-Di-tert-butylphenol	495.5	205.1605	[M-H]-	205.16	NGM_pbdatabase (2.70)	2	HMDB0013816
Methyl 2-(4-chlorophenyl) acetate	43.4	185.0407	[M+H]+	184.09, 125.02, 126.02	NGM_indatabase (2.50)	2	-

Table S8 Continued

Metabolite	RT (min)	Precursor m/z	Ion mode	MS/MS fragment ions (m/z) [a]	Database (source, score) [b]	MSI level [c]	Reference (HMDB ID / PMID) [d]
Pyridine	54.6	80.049	[M+H]+	80.05, 79.02, 55.02, 61.01, 64.00	NGM_pbdatabase (2.20)	2	HMDB0000926
Phytanic acid	34.1	311.2957	[M-H]-	311.30, 311.17, 197.03, 184.02, 226.81	NGM_indatabase (3.99)	1	HMDB0000801
LPC (20:0)	210.1	552.4005	[M+H]+	184.07, 552.40, 104.11, 86.10, 124.99	NGM_indatabase (3.88)	1	HMDB0010390
Procymidone	129.4	282.0093	[M-H]-	282.01, 281.25, 197.03, 165.01, 139.00	NGM_pbdatabase (2.74)	2	HMDB0256790
3,4-Thiophenedicarbonitrile	46.5	135.0022	[M+H]+	135.00, 119.05, 91.05, 134.04, 88.03	NGM_pbdatabase (2.62)	2	-
Tafluprost	360.8	453.2431	[M+H]+	453.24, 233.16, 120.08, 226.88, 86.10	NGM_pbdatabase (2.46)	2	HMDB0015704
7,12-Dioxolithocholic acid	406.6	387.251	[M-H2O+H]+	369.24, 351.23, 370.24, 245.15, 261.18	NGM_indatabase (3.57)	1	HMDB0000447
N-Methyl-D-aspartic acid	301.7	146.0459	[M-H]-	146.05, 116.04, 74.02, 84.05, 98.02	NGM_pbdatabase (2.74)	2	HMDB0002393

Abbreviations: RT, retention time; m/z, mass-to-charge ratio; MS/MS, tandem mass spectrometry; MSI, Metabolomics Standards Initiative; HMDB, Human Metabolome Database; PMID, PubMed Unique Identifier; NGM_indatabase/NGM_pbdatabase, in-house spectral libraries (Biotree).

[a] MS/MS fragment ions listed are the top 3–5 diagnostic ions (after removing isotopic peaks and noise).

[b] Scores in parentheses are MS^2 matching scores from the NGM_indatabase or NGM_pbdatabase.

[c] MSI level 1: confirmed with authentic standard; level 2: putatively identified by spectral matching.

[d] “-” indicates not available in HMDB.

Table S9 Precision results of each Metabolite to be measured in the targeted metabolomic analyses

Metabolite	Within-batch Precision				Between-batch Precision			
	Limit of quantitation	Low	Medium	High	Limit of quantitation	Low	Medium	High
1-Methylguanosine	11.30%	6.54%	7.71%	11.46%	9.52%	3.11%	4.07%	12.13%
N-Methylvaline	11.23%	4.17%	8.79%	12.61%	6.58%	4.81%	3.67%	5.38%
2-Hydroxydesmethylimipramine	11.17%	9.68%	5.83%	9.96%	10.04%	4.27%	3.99%	9.99%
4,5-Dihydro-2-methylthiazole	11.28%	5.00%	5.61%	14.77%	4.96%	3.79%	6.84%	8.99%
SM(d18:1/17:0)	9.75%	9.68%	7.53%	11.49%	6.89%	7.08%	2.91%	7.38%
LPC (20:0)	10.71%	8.38%	8.20%	12.63%	14.36%	4.82%	6.74%	7.03%
2,6-Di-tert-butylphenol	11.29%	6.65%	6.40%	9.86%	8.04%	2.73%	5.06%	13.94%
1,2,2,6,6-Pentamethyl-4-piperidinol	11.26%	4.52%	5.46%	14.19%	7.39%	5.25%	3.89%	7.84%
4-(Chloromethyl)-7-hydroxy-8-methyl-2H-chromen-2-one	9.73%	7.63%	8.09%	14.24%	5.59%	3.05%	6.85%	11.6%
Nonaethylene glycol	8.67%	4.48%	14.30%	13.54%	8.79%	4.19%	6.02%	14.96%
(R)-3-Amino-3-(3-chlorophenyl) propionic acid	11.29%	5.60%	6.57%	14.99%	6.16%	3.74%	6.94%	9.04%
Benzoyleneurea	11.33%	4.23%	7.35%	13.46%	8.58%	6.51%	4.14%	7.37%
Procymidone	11.25%	9.87%	6.89%	14.34%	8.63%	5.90%	4.22%	6.48%

Table S10 Accuracy results of each Metabolite to be measured in the targeted metabolomic analyses

Metabolite	Low	Medium	High
1-Methylguanosine	97.71%	103.48%	97.14%
N-Methylvaline	103.58%	98.34%	97.10%
2-Hydroxydesmethylimipramine	107.61%	97.57%	96.81%
4,5-Dihydro-2-methylthiazole	108.79%	104.94%	100.85%
SM(d18:1/17:0)	113.97%	96.29%	97.16%
LPC (20:0)	112.90%	114.58%	105.30%
2,6-Di-tert-butylphenol	98.74%	101.35%	98.05%
1,2,2,6,6-Pentamethyl-4-piperidinol	114.29%	91.22%	92.70%
4-(Chloromethyl)-7-hydroxy-8-methyl-2H-chromen-2-one	110.07%	112.21%	106.32%
Nonaethylene glycol	106.84%	102.66%	106.93%
(R)-3-Amino-3-(3-chlorophenyl) propionic acid	97.83%	100.36%	97.57%
Benzoyleneurea	98.63%	93.03%	103.83%
Procymidone	111.07%	100.79%	101.20%

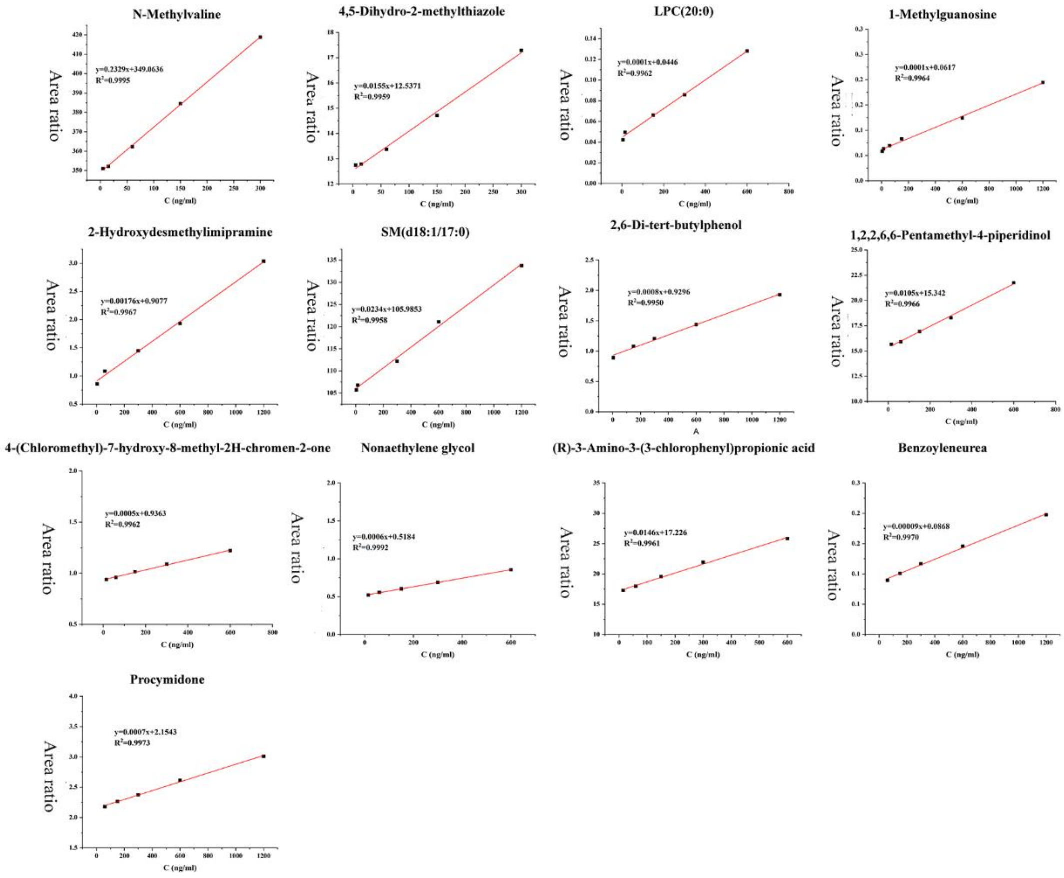

Figure S9 Calibration curves of 13 Metabolites in the targeted metabolomic analyses.

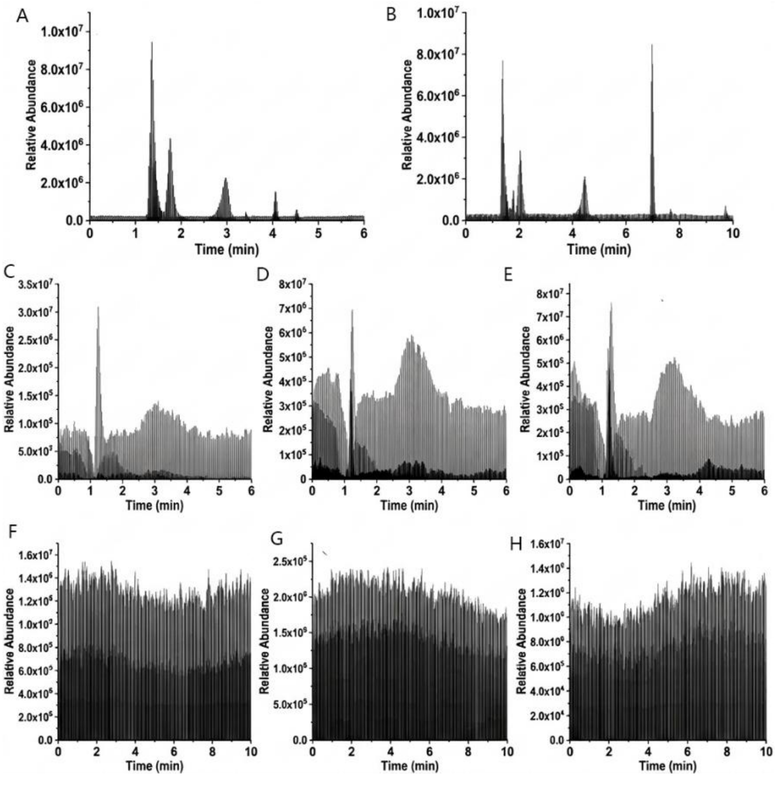

Figure S10 Total ion current profiles of targeted metabolomic analysis under two elution

conditions. Plotted values are integrated TIC intensities at each recorded time point (0.5, 1.5, 2.5, … min) for mixed standards (A, B), healthy controls (C, F), metabolic syndrome (D, G), and pre- metabolic syndrome (E, H) under elution conditions 1 (A, C, D, E) and 2 (B, F, G, H). The consistent intensity patterns across samples confirm stable instrument performance and minimal signal drift during the targeted MRM runs.

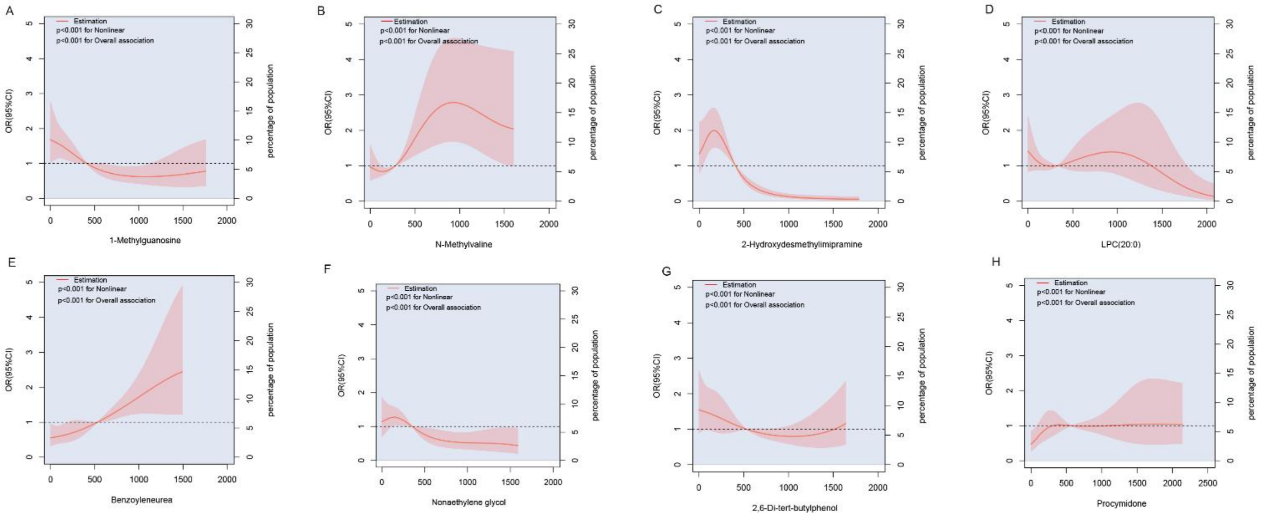

Figure S11 Association Between 8 Metabolites Concentrations and MetS group by using a RCS Regression Model in the validation cohort. RCS: Restricted Cubic Spline; MetS: Metabolic syndrome.

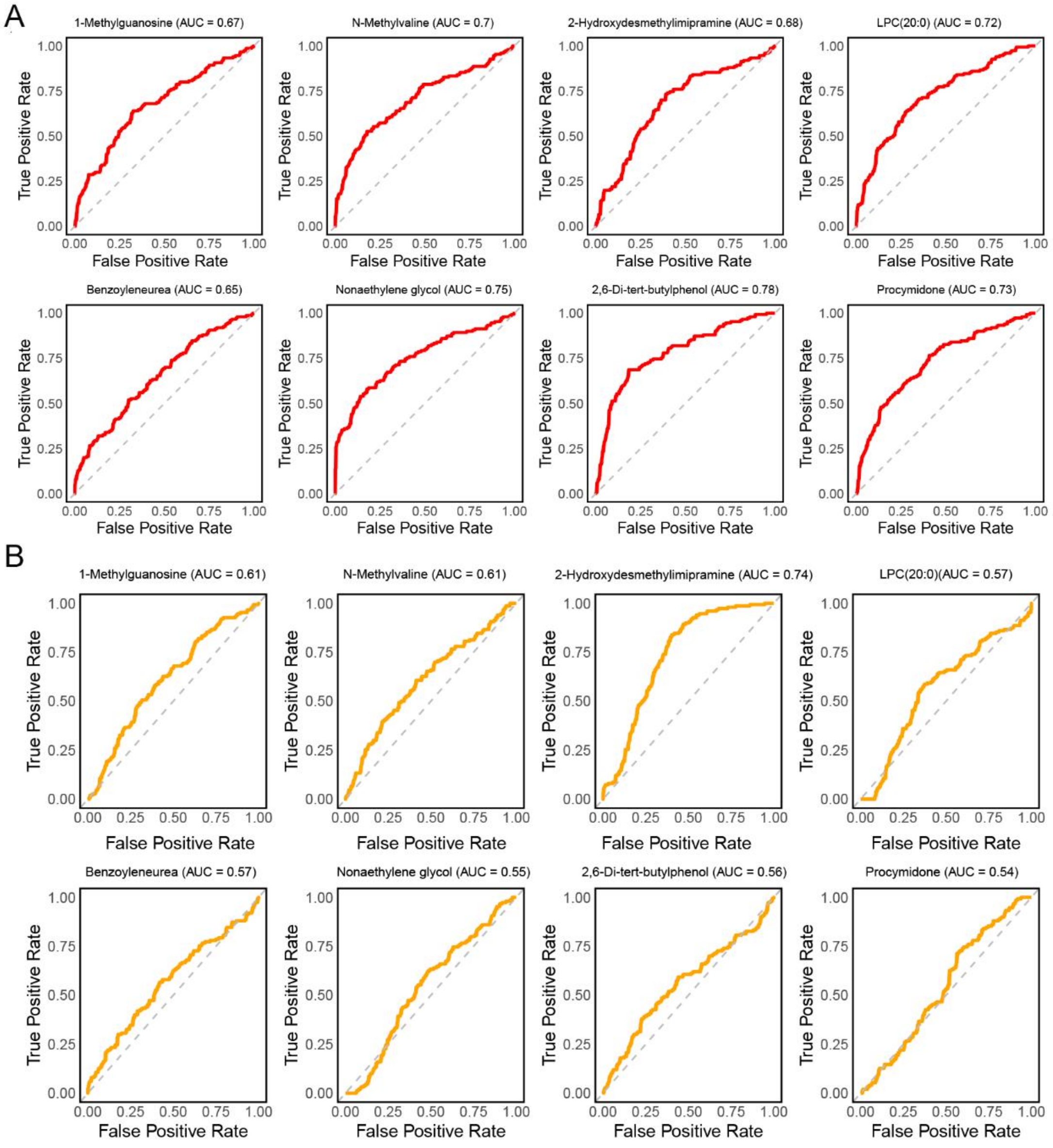

Figure S12 The diagnose performance of 8 Metabolites in the discovery and validation cohort. (A) The ROC curve of 8 Metabolites discriminating MetS in the discovery cohort. (B) The ROC curve of 8 Metabolites discriminating MetS in the validation cohort. MetS: Metabolic syndrome; pre-MetS: Pre- Metabolic syndrome; HC: Health Control; ROC: receiver operating characteristic.

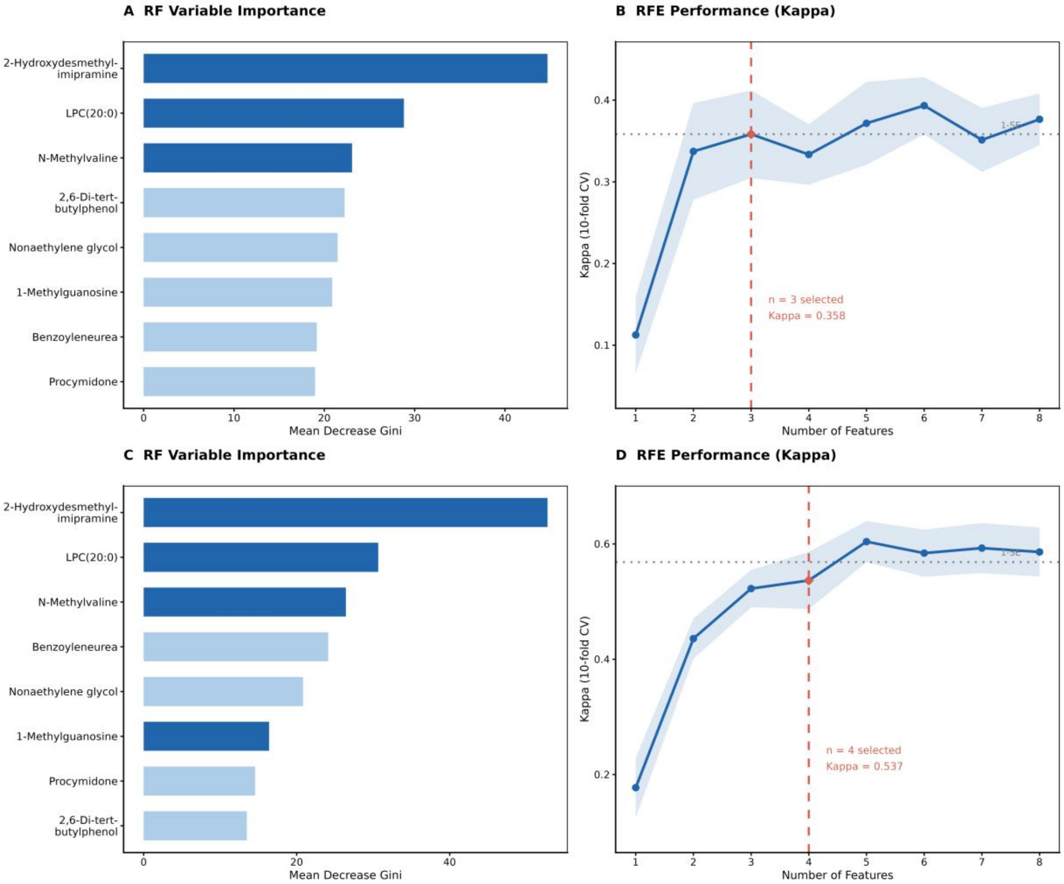

Figure S13 Feature selection results for the two classification groups. (A) Random Forest variable importance for the MetS vs. HC+Pre-MetS group. (B) Recursive Feature Elimination (RFE) Kappa curves for the MetS vs. HC+Pre-MetS group. (C) Random Forest variable importance for the HC vs. MetS +Pre-MetS group. (D) Recursive Feature Elimination (RFE) Kappa curves for the HC vs. MetS +Pre-MetS group. MetS: Metabolic syndrome; pre-MetS: Pre- Metabolic syndrome; HC: Health Control.

Table S11 Machine Learning Model Performance Summary

Group	N_total	Model	AUC	Sensitivity	Specificity	Brier_Score
HC vs. Pre-MetS + MetS	450	SVM	0.789±0.019	0.884±0.087	0.635±0.071	0.177
	450	RF	0.791±0.034	0.820±0.055	0.674±0.058	0.182
	450	Logistic Regression	0.757±0.033	0.873±0.130	0.636±0.112	0.208
HC vs. Pre-MetS + MetS	450	SVM	0.860±0.032	0.874±0.074	0.767±0.072	0.136
	450	RF	0.864±0.023	0.857±0.105	0.759±0.103	0.137
	450	Logistic Regression	0.859±0.030	0.858±0.081	0.767±0.093	0.150

Abbreviations: HC, healthy control; Pre- MetS, pre- metabolic syndrome; MetS, metabolic syndrome; SVM, support vector machine; RF, random forest; AUC, area under the receiver operating characteristic curve.

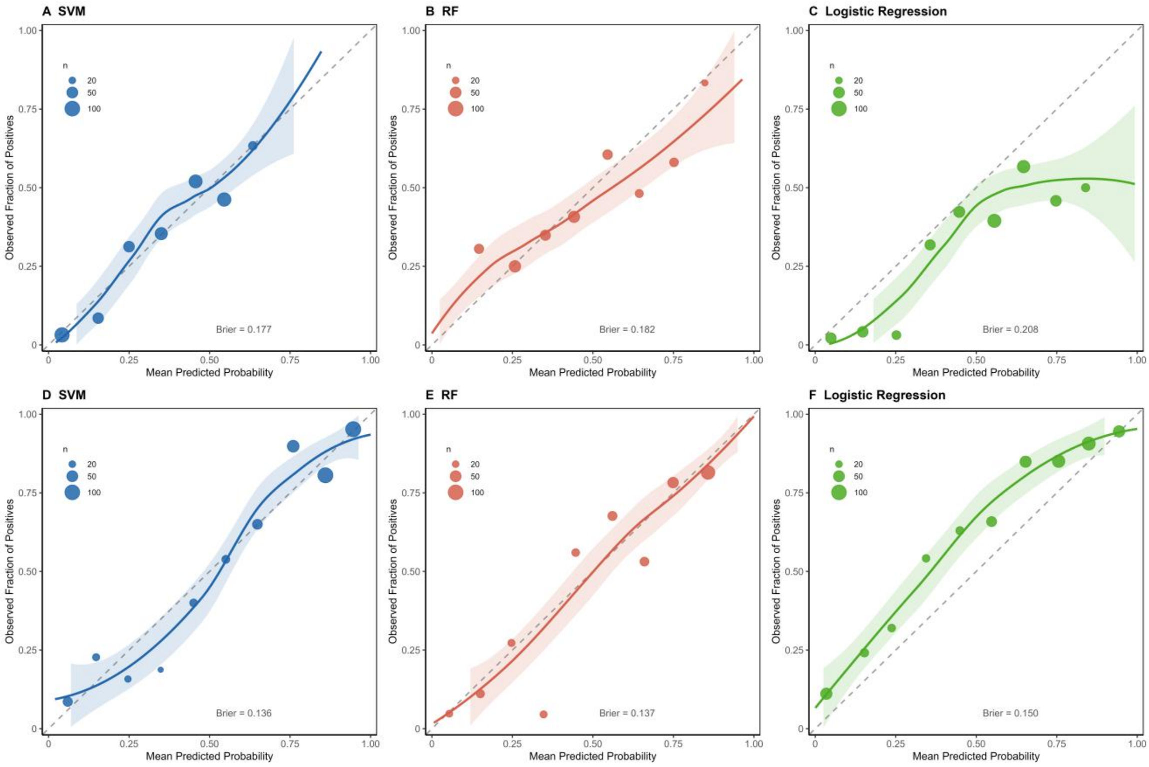

Figure S14 Model calibration curves for the two classification groups. (A) Calibration curves of SVM for the MetS vs. HC+Pre-MetS group. (B) Calibration curves of RF for the MetS vs. HC+Pre-MetS group. (C) Calibration curves of Logistic Regression for the MetS vs. HC+Pre-MetS group. (D) Calibration curves of SVM for the HC vs. Pre-MetS+MetS group. (E) Calibration curves of RF for the HC vs. Pre-MetS+MetS group. (F) Calibration curves of Logistic Regression for the HC vs. Pre-MetS+MetS group.

Table S12 Per-Fold Optimal Hyperparameters

Model	Outer_Fold	C_or_mtry	gamma	Fold_AUC
SVM	1	10	0.1	0.774
	2	10	0.1	0.761
	3	10	0.1	0.791
	4	10	0.1	0.811
	5	100	0.1	0.793
RF	1	1	NA	0.814
	2	1	NA	0.788
	3	2	NA	0.821
	4	2	NA	0.797
	5	1	NA	0.736
SVM	1	10	0.01	0.813
	2	100	0.01	0.847
	3	100	0.01	0.884
	4	100	0.01	0.893
	5	10	0.1	0.862
RF	1	2	NA	0.839
	2	1	NA	0.86
	3	1	NA	0.897
	4	3	NA	0.873
	5	1	NA	0.849

Abbreviations: HC, healthy control; Pre-MetS, Pre-metabolic syndrome; MetS, metabolic syndrome; SVM, support vector machine; RF, random forest; C, cost parameter (SVM); mtry, number of variables randomly sampled as candidates at each split (RF); gamma, kernel coefficient for RBF kernel (SVM); NA, not applicable; AUC, area under the ROC curve.

Table S13 . Baseline characteristics of the urinary metal sub- cohort and comparison with the remaining participants of the parent cohort

Variables	Validation cohort （N=450）	Metal sub- cohort （n=252）	Remaining participants （n=198）	*P* values
Gender，n(%)				<0.05 [a]
Male	201 (44.67)	126 (50.00)	75 (37.88)	
Female	249 (55.33)	126 (50.00)	123 (62.12)	
Age, Median (quartile)	39 (32; 51)	40 (31;52)	39 (32; 50)	0.788 [b]
Smoking, n (%)	108 (24.00)	64 (25.40)	44 (22.22)	0.434 [a]
Alcohol Consumption	102 (22.67)	64 (25.40)	38 (19.19)	0.119 [a]
Waist Circumference, Mean±SD	82.38±10.75	83.26±10.69	81.27±10.76	0.052 [c]
BMI, n (%)				<0.05 [a]
<24.0	197 (43.78)	105 (41.67)	92 (46.46)	
24.0~27.9	149 (33.11)	76 (30.16)	73 (36.87)	
≥28.0	104 (23.11)	71 (28.17)	33 (16.67)	
Hypertension, n (%)	182 (40.44)	109 (43.25)	73 (36.87)	0.171[a]
Hyperglycemia, n (%)	141 (31.33)	86 (34.13)	55 (27.78)	0.149 [a]
HbA1c (%), Mean±SD	5.55±0.80	5.52±0.76	5.58±0.86	0.422 [c]
FPG (mmol/l), Mean±SD	5.60±1.56	5.57±1.50	5.65±1.63	0.577 [c]
TG (mmol/l), Mean±SD	1.46±1.24	1.44±0.97	1.49±1.52	0.676 [c]
TC (mmol/l), Mean±SD	4.19±0.88	4.23±0.92	4.14±0.82	0.280 [c]
HDL-C (mmol/l), Mean±SD	1.26±0.29	1.24±0.28	1.28±0.30	0.157 [c]
LDL-C (mmol/l), Mean±SD	2.53±0.72	2.57±0.77	2.47±0.64	0.123 [c]

Abbreviations: MetS: Metabolic syndrome; pre-MetS: Pre-Metabolic syndrome; HC: Health Control; BMI, body mass index; HbA1c, glycated hemoglobin; FPG, fasting plasma glucose; TG, triglycerides; TC, total cholesterol; HDL-C, high-density lipoprotein cholesterol; LDL-C, low-density lipoprotein cholesterol.

[a] *P* values were obtained from chi-square test.

[b] *P* value was obtained from Kruskal–Wallis rank-sum test.

[c] *P* values were obtained from ttest.

Table S14. Mediation analysis results for metal- metabolite- MetS pathways

Pathway	ACME (95% CI)	ADE (95% CI)	Total Effect (95% CI)	Proportion mediated (%)	*P* (ACME)
Cr→1-Methylguanosine→MetS	0.000 (–0.002, 0.001)	–0.012 (–0.021, –0.003)	–0.013 (–0.022, –0.003)	2.46	0.788
Pb→1-Methylguanosine→MetS	0.002 (0.000, 0.004)	–0.007 (–0.016, 0.003)	–0.005 (–0.014, 0.005)	–35.40	0.080
Cr→N-Methylvaline→MetS	0.000 (–0.001, 0.002)	–0.013 (–0.023, –0.004)	–0.013 (–0.023, –0.003)	–1.74	0.744
Pb→N-Methylvaline→MetS	0.000 (–0.002, 0.002)	–0.005 (–0.014, 0.005)	–0.005 (–0.013, 0.005)	–2.19	0.944
Cd→2-Hydroxydesmethylimipramine→MetS	–0.001(–0.004, 0.000)	–0.005 (–0.014, 0.008)	–0.006 (–0.016, 0.006)	20.53	0.154
Pb→2-Hydroxydesmethylimipramine→MetS	0.001 (0.000, 0.003)	–0.005 (–0.014, 0.004)	–0.004 (–0.013, 0.005)	–24.98	0.148
Pb→LPC (20:0)→MetS	–0.001 (–0.003, 0.000)	–0.004 (–0.013, 0.006)	–0.005 (–0.015, 0.005)	22.86	0.100
Cr→LPC (20:0)→MetS	0.004 (0.001, 0.008)	–0.019 (–0.028, –0.009)	–0.015 (–0.023, –0.006)	–25.50	<0.05
Cu→2,6-Di-tert-butylphenol→MetS	0.000 (–0.005, 0.003)	–0.017 (–0.080, 0.011)	–0.017 (–0.081, 0.012)	1.65	0.802
As→2,6-Di-tert-butylphenol→MetS	0.003 (–0.004, 0.010)	–0.018 (–0.084, 0.025)	–0.016 (–0.080, 0.027)	–16.13	0.470
Mn→Procymidone→MetS	0.000 (–0.009, 0.007)	–0.013 (–0.029, 0.007)	–0.013 (–0.031, 0.006)	3.18	0.836
Hg→Procymidone→MetS	0.004 (0.000, 0.010)	0.003 (–0.009, 0.019)	0.007 (–0.006, 0.025)	53.61	<0.05

Abbreviations: ACME, average causal mediation effect; ADE, average direct effect; CI, confidence interval; MetS, metabolic syndrome; LPC, lysophosphatidylcholine. Metal abbreviations: Cr, chromium; Pb, lead; Cd, cadmium; Cu, copper; As, arsenic; Mn, manganese; Hg, mercury.
